# A Review of Surface Reconstruction and Transformation of 3d Transition‐Metal (oxy)Hydroxides and Spinel‐Type Oxides during the Oxygen Evolution Reaction

**DOI:** 10.1002/smll.202411479

**Published:** 2025-02-07

**Authors:** Biao He, Fan Bai, Priya Jain, Tong Li

**Affiliations:** ^1^ Faculty of Mechanical Engineering Atomic‐scale Characterisation Ruhr‐Universität Bochum Universitätsstraße 150 44801 Bochum Germany

**Keywords:** dissolution, electrolyte effect, facet effect, OER, redeposition, surface amorphization, water electrolysis

## Abstract

Developing efficient and sustainable electrocatalysts for the oxygen evolution reaction (OER) is crucial for advancing energy conversion and storage technologies. 3d transition‐metal (oxy)hydroxides and spinel‐type oxides have emerged as promising candidates due to their structural flexibility, oxygen redox activity, and abundance in earth's crust. However, their OER performance can be changed dynamically during the reaction due to surface reconstruction and transformation. Essentially, multiple elementary processes occur simultaneously, whereby the electrocatalyst surfaces undergo substantial changes during OER. A better understanding of these elementary processes and how they affect the electrocatalytic performance is essential for the OER electrocatalyst design. This review aims to critically assess these processes, including oxidation, surface amorphization, transformation, cation dissolution, redeposition, and facet and electrolyte effects on the OER performance. The review begins with an overview of the electrocatalysts’ structure, redox couples, and common issues associated with electrochemical measurements of 3d transition‐metal (oxy)hydroxides and spinels, followed by recent advancements in understanding the elementary processes involved in OER. The challenges and new perspectives are presented at last, potentially shedding light on advancing the rational design of next‐generation OER electrocatalysts for sustainable energy conversion and storage applications.

## Graphical abstract

3d transition‐metal (oxy)hydroxides and spinel‐type oxides are promising electrocatalysts for the oxygen evolution reaction (OER). However, their electrocatalytic performance alters dynamically during the reaction due to substantial surface changes induced by multiple elementary processes at the electrode/electrolyte interfaces. This review critically assesses these processes, including oxidation, surface amorphization, transformation, cation dissolution, redeposition, and facet and electrolyte effects on the OER performance.

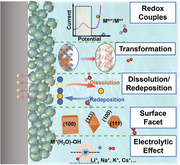

## 1 Introduction

Renewable energy has become a global priority in recent years with the increase in the search for sustainable alternatives to conventional power sources. This transition is driven by the pressing need to mitigate greenhouse gas emissions and reduce reliance on fossil fuels.^[^ ^1^ ^]^ The growing demand for clean energy carriers, i.e., hydrogen, has driven substantial efforts to enhance the target efficiency of water electrolyzers.^[^ ^2^ ^]^ However, one of the challenges lies in developing efficient, affordable, and eco‐friendly catalysts for the oxygen evolution reaction (OER), a critical process in various energy conversion and storage technologies, such as water splitting, fuel cells, and rechargeable metal–air batteries.^[^ ^3^ ^]^ The major issue of OER is that this reaction at the anode of, e.g., water electrolyzers, is kinetically sluggish due to complex four‐electron transfer reactions, making it the bottleneck in the energy conversion systems.^[^ ^4^ ^]^ Nobel‐metal‐based oxides, i.e., IrO_2_ and RuO_2_, are state‐of‐the‐art OER electrocatalysts, but their scarce crustal abundance, high cost, and limited availability limit their large‐scale viability.^[^ ^5^ ^]^ Recent research has focused on improving the efficiency and durability of these materials; however, their performance degradation under operational conditions remains a significant challenge.^[^ ^5^ ^]^ In this context, earth‐abundant and affordable 3d transition‐metal (oxy)hydroxides and spinel‐type oxides have emerged as cheaper alternatives to noble metal oxide electrocatalysts due to their promising OER activity.^[^ ^6^ ^]^ Additionally, the 3d transition‐metal‐based (e.g., Ni and Co) spinel oxides and (oxy)hydroxides offer compositional and structural diversity. Their ease of synthesis and modification allows for the optimizations of physicochemical properties, making them effective OER electrocatalysts.^[^ ^7^ ^]^ Adjustments of the ratios and combinations of various 3d transition cations in the spinel electrocatalysts can further tailor their redox behaviors, enhancing activity and stability toward OER.^[^ ^8^ ^]^

Although numerous successful design strategies of OER electrocatalysts assisted by density function theory (DFT) simulation and cutting‐edge analytical and characterization techniques have been developed,^[^ ^9^ ^]^ what happens at the electrode/electrolyte interface during the reaction remains elusive. Essentially, the electrocatalytic activities are influenced by atomic, electronic, and geometric configurations on the topmost few layers of electrocatalyst surfaces.^[^ ^10^ ^]^ However, the surfaces of OER electrocatalysts often undergo drastic compositional, structural, and morphological changes since multiple elementary processes occur concurrently during the reaction. Such dynamic surface changes alter the activity and stability of electrocatalysts as OER proceeds. This presents difficulties in using descriptors, such as binding energies, metal–oxygen covalency, or, e.g., electron occupancy to predict the activity trend of electrocatalysts during OER since these descriptors estimate the activity of electrocatalysts in the pristine state.^[^ ^11^ ^]^ A detailed understanding of how the elementary processes influence the surface state changes and, thus, electrocatalytic performance is essential for elucidating reaction mechanisms and developing new descriptors for the rational design of efficient OER electrocatalysts.

**Figure 1:**
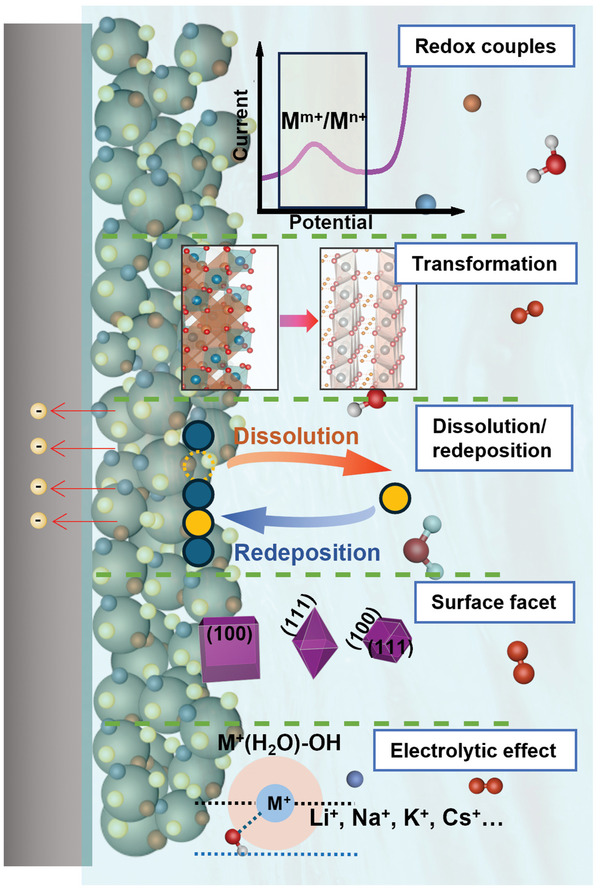
Schematic representation of the elementary process at the electrode/electrolyte interfaces during OER, illustrating the key topics discussed in the review.

Therefore, this review aims to provide a critical assessment of the currently reported elementary processes, i.e., oxidation, surface amorphization, transformation, cation dissolution, and redeposition that occur for the 3d transition‐metal (oxy)hydroxides and spinel‐type oxides (**Figure** **1**). To understand how these processes affect the electrocatalyst surface state evolution, we must establish relationships between the structure of the electrocatalyst surfaces and the OER performance. Thus, we begin with an overview of the structure of spinels and (oxy)hydroxides, followed by a critical discussion of activity and stability measurement protocols for the OER electrocatalysts. We then focus on the effects of the elementary processes, e.g., redox reaction, surface amorphization, transformation, dissolution, and redeposition, on the OER activity and stability. Special attention is also given to how facets and electrolytes affect the activity and stability of electrocatalysts toward OER (Figure 1). Finally, we discuss the remaining challenges and provide new perspectives, which will potentially advance the rational design of efficient OER electrocatalysts for energy storage and conversion applications.

## 2 OER Activity and Stability Measurements of Spinel‐Type Oxides and (Oxy)Hydroxides and Related Issues

Evaluating the OER performance of the electrocatalysts prepared by different research groups worldwide requires benchmarking electrochemical measurement protocols, which have been detailed in previous studies and review papers.^[^ ^12^ ^]^ Here, we mainly focus on the issues associated with measuring the following parameters of 3d transition‐metal spinel‐type oxides and (oxy)hydroxides that cause inconsistencies in assessing the OER performance.

### 2.1 Ohmic Drop Compensation

During the measurement of OER activity, voltage loss occurs mainly due to electrolyte and contact resistances between the working and reference electrodes, resulting in discrepancies between measured and actual potentials.^[^ ^13^ ^]^ For example, different concentrations of KOH are used as electrolytes of OER, and they have different values of uncompensated resistance, *R*_u_ (e.g., ≈6 Ω for 1.0 m KOH and ≈40 Ω for 0.1 m KOH).^[^ ^14^ ^]^ Ohmic drop compensation (*V*_corrected_ = *V*_measured_ − *iR*_u_) is thus performed to eliminate the voltage loss and measure the activity as closely as possible to the actual OER performance of electrocatalysts. *R*_u_ is typically assessed through electrochemical impedance spectroscopy (EIS) or current‐interrupt methods.^[^ ^12^, ^15^ ^]^

**Figure 2:**
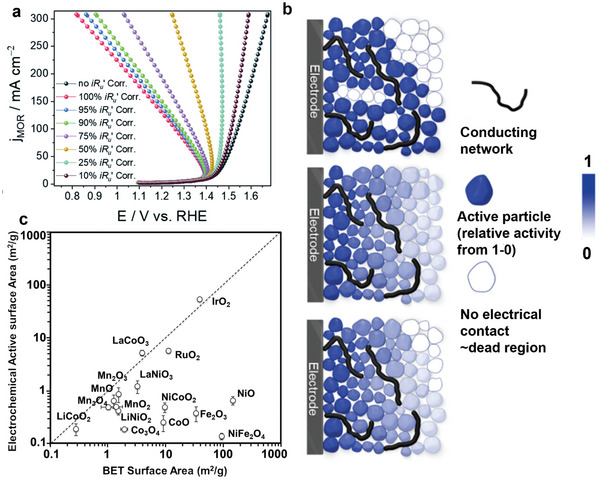
a) The obtained linear sweep voltammetry (LSV) polarization curves with inappropriate *iR*_u_ drop correction due to the high current density and a relatively higher *R*_u_ value. Reproduced (Adapted) with permission.^[^ ^17^ ^]^ Copyright 2016, Royal Society of Chemistry. b) The schematic diagram of the electrochemical utilization of the electrocatalysts influenced by the electrical conductivity, (top) inactive region without electrical contact, (middle) a lower applied overpotential due to the Ohmic loss, (bottom) the combined influence. Reproduced (Adapted) with permission.^[^ ^18^ ^]^ Copyright 2021, American Chemical Society. c) The comparison of the Brunauer–Emmett–Teller (BET) surface area and electrochemical surface area (ECSA) for various metal oxides. Reproduced (Adapted) with permission.^[^ ^19^ ^]^ Copyright 2016, Royal Society of Chemistry.

Generally, a factor is applied to the measured *R*_u_, but the percentage of *iR* drop compensation is inconsistent among the literature, which leads to differences in the reported overpotentials and Tafel slopes, complicating the direct comparison of OER performance among different studies. Researchers often applied 85–95% *iR*_u_ compensation for OER performance measurement.^[^ ^14^, ^16^ ^]^ A 100% *iR*_u_ compensation could help to remove the influence of Ohmic drop. Still, it has been proven impractical at high currents (hundreds of milliamperes) or with electrolytes with high resistance values, potentially leading to “overcorrected” results (see “bend back” polarization curves shown in **Figure** **2**a).^[^ ^17^ ^]^ To avoid overcorrection and ensure accurate reporting of electrocatalyst activity, a 100% *iR*_u_ compensation is recommended but only under suitable current conditions.^[^ ^13^, ^17^ ^]^ Additionally, providing uncompensated polarization curves is suggested for better assessing the impact of *iR*_u_ drop compensation on the reported OER performance (e.g., overpotentials).

### 2.2 Overpotential (*η*)

Overpotential refers to the additional potential required beyond the thermodynamic potential of 1.23 V versus reversible hydrogen electrode (RHE) to drive OER. The extra potential is needed due to the sluggish kinetics of the four‐electron transfer process.^[^ ^20^ ^]^ The overpotentials measured from the linear sweep voltammetry (LSV) curve at a current density of 10 mA cm^−2^ is a commonly used parameter for the OER activity chosen to match the solar‐to‐fuel conversion efficiency of 12.3%.^[^ ^21^ ^]^ However, using overpotentials to indicate the OER activity has some limitations. For instance, for many 3d transition‐metal spinel‐type oxides, pronounced redox peaks appear at potentials near the OER regime of the LSV curves. These peaks contribute to the OER current densities, leading to an inaccurate assessment of the overpotential at 10 mA cm^−2^. To address this issue, it has been proposed to use the overpotential at a higher current density, such as 100 mA cm^−2^, which helps to decouple the redox peak from the OER region and provide a more accurate evaluation of the OER activity.^[^ ^22^ ^]^ However, such high current densities cause excessive bubble formation and detachment of the electrocatalysts from the electrode surface, yielding increased overpotentials.^[^ ^23^ ^]^

Another problem with using overpotentials to compare the OER activity is the discrepancy between the geometric surface area of the electrode and the “true” surface area of the electrocatalyst for current normalization. The current density in the LSV curves is typically calculated based on the geometric surface area. However, this approach does not accurately reflect the actual surface area of electrocatalysts, especially for materials with porous structures, nanoparticles, or thin sheets. These materials have much higher specific surface areas than the geometric areas of the electrode.^[^ ^22^ ^]^ Additionally, the surface area of the OER electrocatalysts changes as OER proceeds due to dynamic surface transformation and reconstruction,^[^ ^24^ ^]^ which further complicates the estimation of surface areas for current normalization. Also, the overpotential measurements are highly dependent on electrode conditions such as catalyst loading and binder types, which causes inconsistent comparisons of overpotentials of electrocatalysts prepared across different labs.^[^ ^25^ ^]^ Thus, overpotential at fixed current densities is probably insufficient to indicate the intrinsic OER activity when the surface area cannot be accurately measured. Universal electrochemical protocols, including standardized catalyst loading, binder types, and electrode setups, are required to facilitate direct comparisons of the overpotentials of OER electrocatalysts.^[^ ^26^ ^]^

### 2.3 Mass Activity, Specific Activity, and Turnover Frequency

The mass activity is defined as the OER current density normalized by the mass loading of the catalyst at a certain overpotential.^[^ ^22^ ^]^ For example, the mass activity of 10.5 A g^−1^ was reported for Co_3−_ *_x_* Cr*_x_* O_4_ spinels at an overpotential of 350 mV, corresponding to a current density of 8.84 mA cm^−2^ at a mass loading of 0.84 mg cm^−2^.^[^ ^27^ ^]^ However, using the mass activity to assess the activity of 3d transition‐metal (oxy)hydroxides and spinel oxides can be tricky due to their low electrical conductivity (Figure 2b).^[^ ^18^ ^]^ Electrocatalysts with poor electrical conductivity have limited electrical contact with each other and the electrode (e.g., glassy carbon). This means some catalyst nanoparticles, especially deep within the electrocatalyst layers, may not effectively participate in the electrochemical reaction. This limited electrical contact increases the electrical resistance of the electrocatalysts, affecting their reaction efficiency and the measured mass activity. Additionally, OER primarily occurs at the surface of the electrocatalysts, meaning a substantial amount of materials beneath the electrocatalyst surfaces may not contribute to the reaction.^[^ ^24a^ ^]^ As a result, mass activity may not accurately reflect the intrinsic activity of electrocatalysts.

To address these limitations, the specific activity of an electrocatalyst, obtained by normalizing the OER current to the surface area of the catalyst, is often used to compare the OER performance. This surface area can be geometric surface area, electrochemical surface area (ECSA), or Brunauer–Emmett–Teller (BET) area, each with its merits and limitations. As previously mentioned, the geometric surface area is suitable for OER current normalization of smooth and planar electrodes.^[^ ^22^ ^]^ However, when dealing with electrodes covered by porous or layered electrocatalysts or nanoparticles,^[^ ^22^ ^]^ using the geometric surface areas yields an overestimated specific activity as these materials have a higher actual surface area than the geometric area.^[^ ^24^ ^]^ In this regard, ECSA is preferred for porous electrocatalysts and nanoparticles as it gives a more accurate estimation of the active surface areas by considering the surface roughness and porosity. ECSA is calculated via the equation ECSA = *C*_dl_/*C*_s_, where *C*_s_ is the specific capacitance of the electrocatalyst per unit area under identical electrolyte conditions, and *C*_dl_ is the double‐layer capacitance determined by EIS or cyclic voltammetry (CV).^[^ ^12a^ ^]^ *C*_s_ varies depending on the material type, electrode potential, and electrolyte composition.^[^ ^24a^ ^]^ For metal oxides, *C*_s_ is 0.015–0.110 mF cm^−2^ in H_2_SO_4_ and 0.022–0.130 mF cm^−2^ in alkaline electrolytes.^[^ ^12^, ^28^ ^]^ However, a constant *C*_s_ value of 0.040 mF cm^−2^ is often used to estimate the surface area of metal oxides in alkaline electrolytes, leading to inaccurate estimation of ECSAs. Additionally, the electrocatalysts undergo surface transformation during OER, whereby newly formed phases, such as (oxy)hydroxides, have different *C*_s_ values compared to the pristine electrocatalysts.^[^ ^29^ ^]^ Therefore, using a constant *C*_s​_ value or directly comparing *C*_dl_ leads to significant inaccuracies in ECSA measurements. An accurate estimation of ECSA requires careful consideration of *C*_s_ and their changes during OER.

Furthermore, previous studies^[^ ^24^, ^30^ ^]^ suggested that the ECSA is only reliable for electrocatalysts with high electrical conductivity. Most oxides exhibit low electrical conductivities (e.g., NiO and CoO), leading to inaccurate *C*_dl_ measurements and low ECSA values (Figure 2c).^[^ ^19^ ^]^ By contrast, BET area measurements via N_2_ adsorption are thought to accurately assess the available surface area.^[^ ^31^ ^]^ It often aligns well with specific electrochemical properties of electrocatalysts, such as the area calculated by analyzing CV peaks and *C*_dl_.^[^ ^32^ ^]^ Thus, researchers sometimes use the BET area to estimate the active surface area of electrocatalysts.^[^ ^12b^ ^]^ However, the BET area is not necessarily electrochemically active since the BET area measurement relies on N_2_ adsorption, which interacts differently with the electrocatalyst surface than water molecules, OH^−^, and other species present during electrochemical reactions. The reconstruction of the electrical double layer at the reaction interface during the OER likely further affects the BET area values.^[^ ^33^ ^]^

Thus, the key problem with using mass and specific activity is that not all electrocatalyst sites contribute to the intrinsic activity. In this regard, turnover frequency (TOF) measures the rate of evolved product molecules (such as O_2_ during OER) per electrochemically active surface site per second, which is a rational indicator of intrinsic activity.^[^ ^12a^ ^]^ To universally apply the TOF for activity comparison, the number of active sites must be accurately determined. Several methods have been suggested to characterize the number of surface active sites of an electrocatalyst.^[^ ^34^ ^]^ For example, one can use the integrated redox peak area to estimate the active sites that directly participate in the redox reactions for some 3d transition‐metal oxide electrocatalysts such as NiO.^[^ ^25^ ^]^ However, for the electrocatalysts without pronounced redox peaks (e.g., Fe), TOF measurements remain challenging due to the difficulties with quantifying active sites.^[^ ^24a^ ^]^ This highlights the need for more reliable characterization techniques or electrochemical protocols to quantify active surface sites for different electrocatalyst materials.

### 2.4 Tafel Slope

The Tafel slope is a crucial parameter for evaluating the OER kinetics and mechanisms. It is derived from the relationship between *η* and logarithm of the current density (*j*) using the Tafel equation^[^ ^35^ ^]^

(1) $$\eta =a+b\cdot \log\left(j\right)$$

In this equation, *a* is the intercept, and *b* is the Tafel slope, calculated as

(2) $$b=\frac{2.303RT}{\alpha nF}$$

where *R* is the gas constant, *T* is the temperature, α is the transfer coefficient, *n* is the number of electrons transferred in the rate‐determining step, and *F* is the Faraday's constant. A lower Tafel slope indicates a rapid increase in current density with increasing overpotential, possibly implying faster charge transfer kinetics in the OER process.^[^ ^35^ ^]^

Potentiodynamic polarization curves such as LSV or CV are often used to calculate the Tafel slope.^[^ ^36^ ^]^ However, such calculation of Tafel slopes can be inaccurate because the Tafel slope depends on the surface coverage of adsorbed intermediate species that varies over time.^[^ ^37^ ^]^ To minimize inaccuracies caused by non‐steady‐state measurements, researchers have employed high‐mass‐transport setups like rotating disk electrode (RDE) and used low catalyst loadings.^[^ ^38^ ^]^ Also, steady‐state techniques such as chronoamperometry (CA) or chronopotentiometry (CP) are employed to measure Tafel slopes.^[^ ^30^, ^39^ ^]^ For example, the Tafel slope of Ni_0.8_Fe_0.2_O*_x_* H*_y_* was determined by performing CP at each current density for several minutes until a constant potential was achieved, ensuring steady‐state conditions.^[^ ^30^ ^]^ Additionally, some studies used both LSV and CA to measure Tafel slopes,^[^ ^37^ ^]^ where the value measured from LSV at a very low scan rate (0.1 mV s^−1^) was similar to that obtained by CA. These approaches enhance the reliability of Tafel slope measurements by promoting steady‐state conditions. Further development of universal, efficient methodologies to measure Tafel slopes under steady‐state conditions remains essential to advance the understanding of charge transfer kinetics during OER.

### 2.5 Electrochemical Stability

Electrochemical stability is often evaluated by performing an accelerated degradation test using CA or CP measurements at a fixed high potential or current density.^[^ ^5^, ^22^ ^]^ Electrocatalysts that exhibit negligible increases in the overpotential or minimal decreases in the current density over extended periods (e.g., more than 10 h) are considered stable.^[^ ^5^, ^22^ ^]^ However, an increase in the overpotential or decrease in the current density during CA and CP measurements does not necessarily indicate that the electrocatalysts are unstable, as these changes can also result from, e.g., gas bubble formation and detachment of electrocatalyst materials from the electrode.^[^ ^40^ ^]^ Specifically, intense bubble generation can weaken the attachment between the electrocatalysts and the electrode substrate at high overpotentials and current densities.^[^ ^23^ ^]^ Therefore, a moderate current density of 10 mA cm^−2^ is suggested for stability estimation, allowing longer‐duration tests (up to 100 h). Alternatively, performing thousands of CV cycles at a high scan rate (e.g., 50 mV s⁻^1^) was recommended for stability evaluation.^[^ ^22^ ^]^ The increase or decrease in current densities after thousands of cycles allows for direct observation of activation or deactivation processes in the electrocatalysts. Notably, the results from CV, CA, and CP stability evaluations are highly dependent on the electrocatalyst loading, and increasing the loading of electrocatalyst materials can significantly enhance the measured lifetime.^[^ ^23^ ^]^ To address this issue, the *S* number (or activity‐stability factor), defined as the ratio between the amounts of evolved oxygen and dissolved metal, was established as a descriptor independent of loading, surface area, or active sites.^[^ ^41^ ^]^ The *S* number provides a precise description of the electrocatalyst's activity versus stability performance; a higher *S* number indicates more excellent stability.

Additionally, CV, CA, CP, and *S* number measurements are performed in electrochemical half‐cells, and the results differ significantly from those measured in proton exchange membrane (PEM) full cells due to variations in electrochemical conditions.^[^ ^42^ ^]^ For example, the lifetime of RuO_2_ measured in a half‐cell was about two orders of magnitude lower than that in a PEM cell.^[^ ^41a^ ^]^ Studies have found that increased overpotentials after stability tests in half‐cells possibly result from the formation of nano‐ and microsized oxygen bubbles rather than electrocatalyst degradation.^[^ ^43^ ^]^ Even when using the RDE technique, oxygen bubbles cannot be entirely removed and may accumulate in the electrocatalysts' pores, blocking the active sites from the electrolyte. Other factors such as local pH, ionomers, supports, impurities, metal ion diffusion rates, and the redeposition of dissolved ions also lead to inconsistent stability measurements between lab‐scale three‐electrode half‐cells and PEM electrolyzers.^[^ ^43^, ^44^ ^]^ Nevertheless, the overall stability trend between the lab‐scale half‐cells and PEM is believed to be transferable, although the exact values may differ dramatically.^[^ ^45^ ^]^ For instance, the electrocatalysts with a lower *S* number measured in lab‐scale cells than RuO_2_ are considered to be insufficiently stable in a PEM electrolyzer.^[^ ^45^ ^]^

## 3 Elementary Processes and Their Effects on OER Performance

### 3.1 Structure of Spinel‐Type Oxides, (Oxy)Hydroxides, and Layered Double Hydroxides

#### 3.1.1 Spinel‐Type Oxides

**Figure 3:**
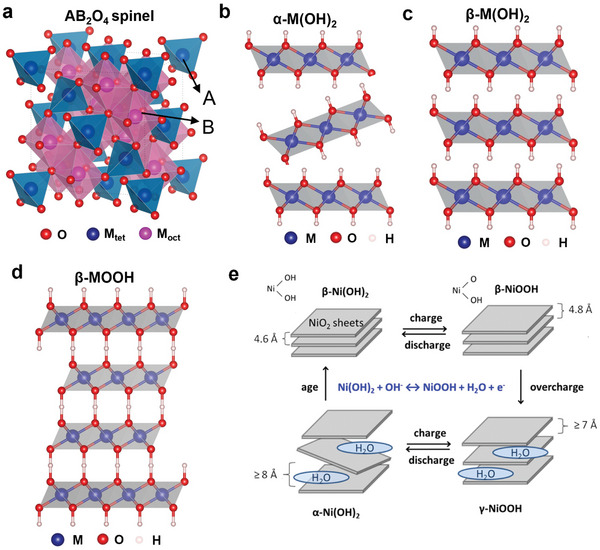
Crystal structure of a) spinel, b) α‐M(OH)_2_ (*R*3__*m*), c) β‐M(OH)_2_ (*P*3*m*1), d) β‐MOOH (*P*3*m*1) and e) Bode plot depicting the phase transition routes in α‐Ni(OH)_2_ with applied bias. Reproduced (Adapted) with permission.^[^ ^59^ ^]^ Copyright 2015, ACS Publications.

Spinel‐type oxides with a chemical formula of AB_2_O_4_ are classified into three types: normal, inverse, and complex spinels, depending on several factors such as A and B cation radius, size of the interstitial sites, ionic charge, and crystal‐field stabilization energy.^[^ ^46^ ^]^ In normal spinels, such as MgAl_2_O_4_, A^2+^ occupies tetrahedral (T_d_) sites, and B^3+^ occupies [O_h_] sites within a face‐centered cubic oxygen lattice (**Figure** **3**a), resulting in the formula (A^2+^)_Td_[B^3+^]_Oh_O_4_. By contrast, inverse spinels, such as NiFe_2_O_4_, have A^2+^ cations occupying O_h_ sites, and B^3+^ cations take up both T_d_ and O_h_ sites, given by the formula (B^3+^)_Td_[A^2+^B^3+^]_Oh_O_4_. Complex spinels exhibit a mixed distribution, with both A^2+^ and B^3+^ cations partially occupying T_d_ and O_h_ sites, represented as (A^2+^_1−_ *_λ_* B^3+^ *_λ_*)_Td_[A^2+^ *_λ_* B^3+^_2−_ *_λ_*]_Oh_O_4_. The degree of inversion depends on the nature of the elements involved, their Coulombic interactions, and the synthesis conditions.^[^ ^8b^ ^]^ Due to their ability to accommodate various transition‐metal cations, spinels offer versatile structural flexibility for tuning electrocatalytic activity, leading spinels to exhibit higher OER activities than state‐of‐the‐art electrocatalysts.^[^ ^8b^ ^]^ The synergistic effect of multiple metals within spinel‐based systems on electrochemical performance has also been explored.^[^ ^47^ ^]^ For instance, Ru and Ir in noble metal oxides serve as key elements to enhance overall electrocatalytic performance, especially in acidic media.^[^ ^48^ ^]^ 3d transition metals such as Fe, Co, and Ni with excellent electrical conductivity are active for high electrocatalytic activity in alkaline environments.^[^ ^49^ ^]^ In comparison, Cr, Zn, and Al might not be electrocatalytic active compared to Fe, Co, and Ni. However, their rapid leaching rates during the reactions create vacancies and defects that significantly enhance activity due to the increased active surface areas.^[^ ^50^ ^]^ For Mo and W, they regulate electronic structures in, e.g., FeCoMoW high entropy oxides, inducing long‐term stability toward OER even at high potentials.^[^ ^51^ ^]^ In addition to tailored multimetal compositions, fundamental principles such as metal–oxygen covalency,^[^ ^52^ ^]^ *E*_g_ filling theory,^[^ ^53^ ^]^ or amounts of octahedral sites^[^ ^54^ ^]^ on the surface of electrocatalysts have been used to design and optimize spinel‐type oxides for OER. Also, other strategies such as doping,^[^ ^55^ ^]^ creating oxygen vacancies,^[^ ^56^ ^]^ tuning morphology,^[^ ^57^ ^]^ and facet engineering^[^ ^58^ ^]^ have been employed to enhance the intrinsic activities of spinel electrocatalysts. These design strategies have been thoroughly reviewed in several papers.^[^ ^7^, ^8^ ^]^ Despite the success of these concepts, the spinel‐type oxides transform oxyhydroxide layers, which could increase conductivity, stabilize active sites, and aid intermediate species adsorption, as will be elaborated in the following section.

#### 3.1.2 Layered Double Hydroxides

Layered double hydroxides (LDHs) are a class of 2D materials derived from hydrotalcite‐like divalent metal hydroxides with the general chemical formula [M_1−_ *_x_* ^2+^M*_x_* ^3+^(OH)_2_]*^x^* ^+^(A*^n^* ^−^)*_x_*_/_ *_n_* ·*m*H_2_O,^[^ ^60^ ^]^ where M represents divalent and trivalent transition‐metal cations, and A is the intercalated anion. The structure of LDHs consists of MO_6_ octahedra arranged in edge‐sharing 2D layers (Figure 3b), forming a brucite‐like M^II^(OH)_2_ base with partial substitution of M^III^ cations to create positively charged layers, balanced by intercalated anions such as CO_3_^2−^, NO_3_^−^, or Cl^−^.^[^ ^60^ ^]^ The lattice parameters of LDHs vary depending on the metal composition and interlayer anions. For instance, Ni‐based LDHs have lattice constant *a* of 3.08 Å and the interslab distance of ≥8 Å.^[^ ^61^ ^]^ The Ni‐based LDHs undergo structural transformations under various electrochemical conditions, transforming among four phases: α‐Ni(OH)_2_, β‐Ni(OH)_2_, γ‐NiOOH, and β‐NiOOH (see Bode plot in Figure 3e).^[^ ^62^ ^]^ These phases share a layered architecture of edge‐sharing NiO_6_ octahedra but differ in their crystallinity, interslab distance, and electrochemical properties, as summarized in **Table** **1**. The α‐Ni(OH)_2_ phase with a relatively poor crystallinity exhibits higher electrochemical reactivity. It converts into the more stable β‐Ni(OH)_2_ phase over time, especially during cycling in alkaline media.^[^ ^61^ ^]^ The β‐Ni(OH)_2_ has a well‐ordered hexagonal brucite‐like structure with lattice constants *a* and *c* of 3.11 and 4.62 Å, respectively (Figure 3c),^[^ ^63^ ^]^ which offers better electrochemical stability and cycling performance due to its low water content. Upon charging, β‐Ni(OH) is oxidized to β‐NiOOH that can convert to γ‐NiOOH with a significant increase in the interslab distance ranging from 4.8 to ≥7 Å at overcharging conditions.^[^ ^64^ ^]^ Alternatively, the α‐Ni(OH)_2_ phase can transform to γ‐NiOOH reversibly upon charging and discharging (Figure 3e). The α‐Ni(OH)_2_ to γ‐NiOOH transition results in a more disordered and less defined structure compared to the β‐Ni(OH)_2_ to β‐NiOOH transition.^[^ ^65^ ^]^

**Table 1:** Crystal structure and properties of Ni hydroxides and oxyhydroxides.

Compound/phase	Crystal structure	Lattice constants	Space group	Key properties	Refs.
α‐Ni(OH)_2_	Hexagonal, brucite‐like with intercalated anions and water	*a* ≈ 3.07 Å *c* ≈ 23.2 Å with an interslab distance ≈ 7–9 Å	*R*3__*m*	– Poor crystallinity – Higher electrochemical reactivity – Tends to convert to β‐Ni(OH)_2_ over time in alkaline solution – Exhibits layered structure with larger interlayer spacing due to intercalated water and anions	[74]
Layered double hydroxide (LDH)	Hexagonal, brucite‐like with substituted cations and anions			– Tunable properties via metal composition and interlayer anions – Higher electrochemical reactivity – Suitable for catalysis and energy storage applications – Structure consists of positively charged layers balanced by interlayer anions – Exhibits ion‐exchange and intercalation capabilities	
β‐Ni(OH)_2_	Hexagonal, brucite‐like	*a* = 3.11 Å *c* = 4.62 Å	*P*3*m*1	– Well‐ordered structure – High crystallinity – Better electrochemical stability and cycling performance – Lower interlayer spacing compared to α‐Ni(OH)_2_ – More stable in alkaline solutions	[75]
β‐NiOOH	Hexagonal	*a* = 2.81 Å *c* = 4.84 Å	*P*3*m*1	– Formed by oxidation of β‐Ni(OH)_2_ – More crystalline and stable – Higher equilibrium potential – Better electrochemical reversibility – Retains similar structure to β‐Ni(OH)_2_ with slight lattice parameter changes due to oxidation	[76]
γ‐NiOOH	Rhombohedral, disordered with intercalated water and cations	*a* = 2.82 Å *c* = 20.65 Å with an interslab distance ≈ 7 Å	*R*3__*m*	– Formed under more oxidative conditions – Disordered structure with expanded layers – Intercalation of water and alkali ions increases interlayer spacing – Higher electrical conductivity – Increased capacity and intercalation properties – Lower discharge potential and reduced stability compared to β‐NiOOH	[74]

Typically, LDHs show OER overpotentials of around ≈200 mV, lower than those of benchmark OER catalysts like RuO_2_ (≈280 mV at 10 mA g^−1^) and IrO_2_ (≈290 mV at 10 mA g^−1^).^[^ ^66^ ^]^ Incorporating other 3d transition‐metal cations with high electrophilicity, such as Fe,^[^ ^67^ ^]^ Mn,^[^ ^68^ ^]^ and Cr,^[^ ^69^ ^]^ into the LDH lattice improves the OER activity by lowering overpotentials and increasing stability.^[^ ^70^ ^]^ Additionally, tuning the metal cations in the host layers and changing the interlayer cations or anions can improve the electrocatalytic performance of LDHs.^[^ ^71^ ^]^ For example, benzoate‐anion‐intercalated layered Ni(OH)_2_ induces interlayer expansion, accommodating more electrolyte ions and facilitating diffusion, thus providing a high electrocatalytic activity.^[^ ^61^, ^71^ ^]^ Besides anion intercalation, the introduction of cations, for example, TMA^+^, can interact with the active oxygen species during deprotonation and disrupt the hydrogen bond network of the interfacial water with the Ni(Fe)OOH catalysts, thus accelerating the OER efficiency.^[^ ^72^ ^]^ Another noteworthy characteristic of LDHs is their promising electrical conductivity due to electron hopping mechanisms and the exchange of oxidation states between different metal sites.^[^ ^73^ ^]^ For instance, after doping NiFe LDH with high oxidation state cations such as Cr, Ru, Ce, and V, the strong electronic interaction between Cr and NiFe sites during OER was achieved, giving rise to a higher charge transfer efficiency.^[^ ^73^ ^]^

#### 3.1.3 Metal Oxyhydroxide (MOOH)

The 2D‐layered MOOHs (where M = Fe, Ni, Co, Mn, V) consist of edge‐sharing MO_6_ octahedra, which stack to form a 2D structure.^[^ ^77^ ^]^ These materials are primarily classified as β‐ and γ‐MOOH, each with unique properties that influence their OER performance (Table 1).^[^ ^78^ ^]^ In Ni‐based systems, β‐NiOOH has a hexagonal structure (*P*3*m*1; *a* = 2.82 Å and *c* = 4.84 Å) (Figure 3d). β‐NiOOH is crystalline, stable, and offers high electrochemical reversibility. By contrast, γ‐NiOOH has a disordered rhombohedral structure (space group: *R*3__*m*), with a much larger interslab distance of ≈7 Å due to the intercalation of water and alkali ions. Some studies reported that β‐NiOOH is active toward OER,^[^ ^79^ ^]^ while others proposed that γ‐NiOOH is more active due to the formation of catalytically active intermediates with enhanced electrical conductivity.^[^ ^80^ ^]^ Among 3d transition‐metal‐based oxyhydroxides, NiOOH and CoOOH have been shown to outperform Fe‐, Cr‐, and Mn‐based oxyhydroxides.^[^ ^81^ ^]^ Lim et al. found that the OER activity sequence follows NiO(OH) > CoO(OH) > FeO(OH) > CrO(OH) > MnO(OH).^[^ ^81^ ^]^ Mixed CoNi oxyhydroxide exhibits an improved OER performance with an overpotential of 279 mV at 10 mA cm^−2^ and a Tafel slope of 62 mV dec^−1^ in alkaline solutions.^[^ ^82^ ^]^ This suggests the importance of tuning oxyhydroxides' composition and electronic structure in improving the OER performance. However, determining the active sites in oxyhydroxides remains a notorious challenge, which requires forthcoming experimental and theoretical studies.

### 3.2 Redox Couples of 3d Transition Metals in Spinel and (Oxy)Hydroxides

3d transition‐metal (oxy)hydroxides and spinel‐type oxides undergo redox processes before OER. The redox states of the 3d transition‐metal cations can influence the binding energies and bond strengths of intermediates such as MO, MOH, and MOOH. The redox state changes also affect the availability of electrons necessary for breaking or forming chemical bonds. Therefore, a thorough understanding of the redox behaviors of different 3d transition metals, particularly the potentials at which these processes occur and their impact on OER activity, is crucial for predicting the dominant metal sites for the redox reactions before OER. We have summarized the redox peak positions of Ni, Co, and Cr in **Table** **2** based on various reported catalyst materials and electrochemical conditions.^[^ ^83^ ^]^

**Table 2:** Summary of anodic and cathodic potential peak center of some Ni‐, Co‐, and Cr‐based catalysts during CV with different conditions.

Element	Catalyst	Electrolyte	Scan rate [mV s^−1^]	Anodic potential center I	Cathodic potential center II	Refs.
Ni	Ni(OH)_2_	2 m KOH	–	≈0.45 V versus Ag/AgCl	≈0.3 V versus Ag/AgCl	[107]
	Ni_0.82_Fe_0.18_OOH	2 m KOH	–	≈0.48 V versus Ag/AgCl	≈0.35 V versus Ag/AgCl	[107]
	40% Ni on Raney Ni	0.1 m KOH	20	≈1.45 V versus RHE	≈1.3 V versus RHE	[108]
	40% Co on Raney Ni	0.1 m KOH	20	≈1.25 V versus RHE	≈1.15 V versus RHE	[108]
	40% Fe on Raney Ni	0.1 m KOH	20	≈1.45 V versus RHE	≈1.4 V versus RHE	[108]
	(Ni,Fe)OOH	0.1 m KOH	10	≈1.5 V versus RHE	≈1.41 V versus RHE	[84d]
	Ni_100−_ *_x_* Fe*_x_* OH	0.1 m KOH	100	≈1.4 V versus RHE (redshift with *X* increase)	≈1.35 V versus RHE (redshift with *X* increase)	[86]
	Ni_100−_ *_x_* Fe*_x_* OH	0.1 m KOH	10	≈1.4 V versus RHE (redshift with *X* increase)	≈1.35 V versus RHE (redshift with *X* increase)	[86]
	Ni(OH)_2_	1 m KOH	–	≈0.45 V versus Hg/HgO	≈0.35 V versus Hg/HgO	[104]
	Ni_100−_ *_x_*‐Fe*_x_* films	0.1 m KOH	10	≈0.5 V versus Hg/HgO (redshift with *X* increase)	≈0.41 V versus Hg/HgO (redshift with *X* increase)	[109]
	NiFeO*_x_* F*_y_* nanosheets	1 m KOH	50	≈1.4 V versus RHE	≈1.3V versus RHE	[105a]
	F─NiFeO*_x_* F*_y_* nanosheets	1 m KOH	50	≈1.45 V versus RHE	≈1.35 V versus RHE	[105a]
	Ni(OH)_2_	1 m KOH	50	≈1.42 V versus RHE	≈1.3 V versus RHE	[84b]
	NiCo LDH	1 m KOH	50	≈1.4 V versus RHE	≈1.25 V versus RHE	[84b]
	NiMn LDH	1 m KOH	50	≈1.42 V versus RHE	≈1.3 V versus RHE	[84b]
	NiFe LDH	1 m KOH	50	≈1.45 V versus RHE	≈1.35 V versus RHE	[84b]
	ZnCo_1.2_Ni_0.8_O_4_	1 m KOH	10	≈1.4 V versus RHE	≈1.3 V versus RHE	[110]
	NiCo_2_O_4_	1 m KOH	100	≈1.4 V versus RHE	≈1.3 V versus RHE	[111]
	LiNiO_2_	1 m KOH	10	≈1.45 V versus RHE	≈1.35 V versus RHE	[112]
Co	Co_3_O_4_	2 m KOH	10	≈1.2 V versus RHE; ≈1.4 V versus RHE	≈1.5 V versus RHE; ≈1.45 V versus RHE	[90]
	Co(OH)_2_	1 m KOH	50	1.2 V versus RHE; 1.45 V versus RHE	≈1.1 V versus RHE; ≈1.5 V versus RHE	[84b]
	CoCo LDH	1 m KOH	50	1.22 V versus RHE; ≈1.5 V versus RHE	≈1.19 V versus RHE; ≈1.5 V versus RHE	[84b]
	CoFe LDH	1 m KOH	50	≈1.22 V versus RHE; ≈1.5 V versus RHE	≈1.19 V versus RHE; ≈1.5 V versus RHE	[84b]
	CoMn LDH	1 m KOH	50	Broad peak 1.2–1.5 V	Broad peak 1.2–1.5 V	[84b]
	Co_3_O_4_	1 m KOH	2	≈1.2 V versus RHE; ≈1.45 V versus RHE	≈1.2 V versus RHE; ≈1.4 V versus RHE	[113]
	NiMn─Co_3_O_4_	1 m KOH	2	≈1.2 V versus RHE; ≈1.42 V versus RHE	≈1.2 V versus RHE; ≈1.38 V versus RHE	[113]
	Co_3_O_4_	0.1 m KPI	50	≈1.4 V versus RHE; ≈1.4 V versus RHE	≈1.55 V versus RHE; ≈1.55 V versus RHE	[88]
	CoOOH	0.1 m KPI	50	≈1.4 V versus RHE; ≈1.6 V versus RHE	≈1.4 V versus RHE; ≈1.6 V versus RHE	[88]
	Co_3_O_4_ nanocube	0.1 m KOH	50	≈1.25 V versus RHE; ≈1.45 V versus RHE	≈1.25 V versus RHE; ≈1.4 V versus RHE	[114]
	CoCr_2_O_4_	1 m KOH	10	≈1.15 V versus RHE; ≈1.45 V versus RHE	≈1.1 V versus RHE; ≈1.45 V versus RHE	[92a]
	CoO*_x_* (OH)*_y_*	0.1 m KOH	100	≈1.12 V versus RHE; ≈1. 5 V versus RHE	≈1.1 V versus RHE; ≈1.5 V versus RHE	[87]
	Co microelectrode	0.1 m KOD	10	≈1.2 V versus RHE; ≈1. 5 V versus RHE	≈1.1 V versus RHE; ≈1.5 V versus RHE	[115]
	β‐Co(OH)_2_	0.1 m KOH	Between 0.25 and 1000	≈1.25 V versus RHE; ≈1. 55 V versus RHE	≈1.2 V versus RHE; ≈1.5 V versus RHE	[116]
	ZnCo_2_O_4_ and Co_3_O_4_	0.1 m KOH	50	≈1.2 V versus RHE; ≈1. 5 V versus RHE	≈1.15 V versus RHE; ≈1.45 V versus RHE	[117]
	CoAl_2_O_4_	1 m KOH	10	≈1.4 V versus RHE	≈1.37 V versus RHE	[97c]
	CoFe_0.25_Al_1.75_O_4_	1 m KOH	10	First cycle ≈1.32 V and second 1.18 versus RHE	≈1.15 versus RHE	[97c]
	Co_2_FeO_4_ nanoparticle	1 m KOH	10	≈1.19 V versus RHE; ≈1.48 V versus RHE	≈1.1 V versus RHE; ≈1.44 V versus RHE	[31]
	V_O_─Co_3_O_4_	1 m KOH	10	≈1.25 V versus RHE; ≈1. 47 V versus RHE	≈1.4 V versus RHE	[103a]
Cr	CoCr_2_O_4_	1 m KOH	10	≈1.3 V versus RHE	≈1.25 V versus RHE	[92a]
	ZnCr_2_O_4_	1 m KOH	2	≈1.3 V versus RHE	≈1.24 V versus RHE	[92b]
	CoCr_2_O_4_	1 m KOH	10	≈1.3 V versus RHE	≈1.25 V versus RHE	[118]

**Figure 4:**
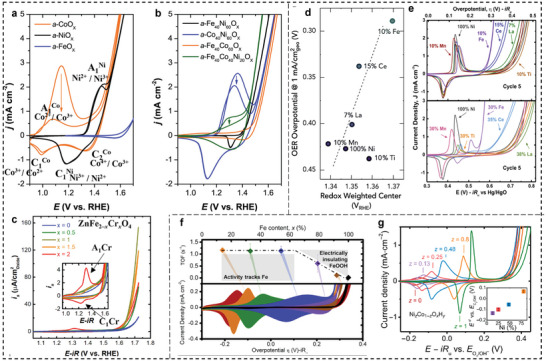
Cyclic voltammogram data for a) single and b) mixed metal oxides were collected at a scan rate of 10 mV s^−1^ in 0.1 m KOH, arrows indicate the progression of scans. Reproduced (Adapted) with permission.^[^ ^84b^ ^]^ Copyright 2017, American Chemical Society. c) CV curves for ZnFe_2−_ *_x_* Cr*_x_* O_4_ with *x* = 0–2, where *x* = 2 shows ZnCr_2_O_4_ with redox peak at 1.3 V corresponding to Cr^3+^/^4+^ redox couple. Reproduced (Adapted) with permission.^[^ ^93b^ ^]^ Copyright 2018, American Chemical Society. d) The relationship between the OER activity and the weighted center position of the Ni redox peaks for metal‐doped nickel hydroxides, with the scan rate of 20 mV s^−1^. Reproduced (Adapted) with permission.^[^ ^95^ ^]^ Copyright 2018, Elsevier. e) CV cycle of Ni_1−_ *_z_* M*_z_* O*_x_* H*_y_* films, where M is the metal cation (Ce, La, Mn, Ti, and Fe), in Fe‐free 1 m KOH at 20 mV s^−1^. Reproduced (Adapted) with permission. Copyright 2021, American Chemical Society. f) CV curve of Co_1−_ *_x_* Fe*_x_* (OOH) showing systematic anodic shift of the Co^2+/3+^ peak with increasing Fe content. Reproduced (Adapted) with permission.^[^ ^99^ ^]^ Copyright 2015, American Chemical Society. g) CV cycle of Ni(Co)O*_x_* H*_y_* (10 mV s^−1^ in 1 m KOH) as a function of Ni content. Reproduced (Adapted) with permission.^[^ ^101^ ^]^ Copyright 2019, Springer Nature.

The redox process of Ni is centered at ≈1.4 V versus RHE, which is often assigned to the Ni^2+^/Ni^3+^ transition.^[^ ^84^ ^]^ This transition may arise from hydroxide‐mediated oxidation and deprotonation between Ni^2+^(OH)_2_ and Ni^3+^OOH.^[^ ^84^ ^]^ Assigning these redox couples is challenging, and it requires operando measurements, such as X‐ray absorption spectroscopy (XAS), to confirm the oxidation states at varying potentials. For instance, operando XAS results in previous work^[^ ^85^ ^]^ indicated that the oxidation state of Ni can exceed +3, reaching up to +3.6, which suggests the potential formation of Ni^4+^ before OER. Also, Strasser and co‐workers have consistently found that up to 25% of Ni sites reach an oxidation state of +4 in the Ni─Fe oxyhydroxide electrocatalyst.^[^ ^86^ ^]^ For Co‐based spinels and (oxy)hydroxides, two pairs of redox peaks are typically observed at 1.1–1.2 and 1.4–1.5 V versus RHE, associated with the Co^2+^/Co^2+,3+^, and Co^2+,3+^/Co^3+^ transitions during hydroxide‐mediated deprotonation and protonation processes (see **Figure** **4**a,b).^[^ ^83b^ ^]^ Some studies assigned the redox peak between 1.5 and 1.6 V versus RHE to the Co^3+^/Co^4+^ transition.^[^ ^87^ ^]^ At the same time, other researchers have questioned the existence of Co^4+^ as K‐edge shifts observed in operando XAS measurements may not solely result from an increased Co oxidation state.^[^ ^88^ ^]^ Instead, the K‐edge shift can also be induced by, e.g., Co─O bond contraction due to the formation of an oxyl‐adsorbate on the catalyst surface, causing charge reorganization in the 3d orbitals of the Co^3+^ ions during OER.^[^ ^88^ ^]^ These findings imply that the formation of Co^4+^ might be unlikely; the in situ formation of a 3D cross‐linked CoO*_x_* (OH)*_x_* with di‐µ‐oxo‐bridged Co^3+^ species is responsible for the observed redox behavior.^[^ ^88^ ^]^ On the other hand, a recent study using surface interrogation scanning electrochemical microscopy suggests that mixed‐valence states of Co^3+^/Co^4+^ might be present on cobalt phosphate^[^ ^89^ ^]^ and Co spinels,^[^ ^90^ ^]^ acting as the active species. Thus, the presence of high oxidation states like Ni^4+^ or Co^4+^ during OER remains debatable. Detecting and determining such high oxidation states is tricky, especially during electrochemical cycling. Consequently, it remains unclear whether these high Ni and Co oxidation states (+4) are the key to the enhanced OER activity of Ni‐ and Co‐based electrocatalysts.^[^ ^91^ ^]^ For Cr‐based spinels, the CV curves of, e.g., CoCr_2_O_4_ and ZnCr_2_O_4_ show redox peaks around 1.3 V versus RHE corresponding to the Cr^3+^/Cr^4+^ transition (Figure 4c).^[^ ^92^ ^]^ In comparison, Mn‐containing spinels display redox‐featureless CV curves.^[^ ^93^ ^]^ The absence of redox peaks is attributed to their pseudocapacitive characteristics, which are well‐recognized in supercapacitor studies.^[^ ^94^ ^]^

Similarly, Fe also does not exhibit pronounced redox peaks in the CV curves of Fe‐containing spinels.^[^ ^84a^ ^]^ On the other hand, Fe can influence the redox behavior of other 3d transition‐metal cations, e.g., by shifting the potentials of Ni^2+^/Ni^3+^ or Co^3+^/Co^3+,4+^ redox transitions to higher values, as exemplified by Ni‐Fe oxide thin films shown in Figure 4b.^[^ ^95^, ^96^ ^]^ Essentially, the role of Fe doping in the Ni‐ and Co‐based (oxy)hydroxides has been the subject of debate for a decade,^[^ ^97^ ^]^ which has been carefully reviewed.^[^ ^67^, ^98^ ^]^ The key conflicts are if Fe is 1) an indirect active site, 2) active for OER, or 3) has a synergistic effect with Co and Ni for enhancing OER activity. Specifically, one perspective is that Fe modifies the redox behavior of Ni and Co without being directly active toward OER. Previous studies^[^ ^14^, ^95^, ^96^ ^]^ have suggested that incorporating Fe^3+^ (which has a lower p*K*_a_ value and higher electronegativity, Figure 4d,e) into Ni(OH)_2_ shifted the redox potential of Ni to a higher value and decreased the overpotential. Such a shift was associated with the decrease in the energy of the antibonding states in M─O bonds, which lowered the energy related to the redox of M‐ligand bonds (M─OH and M─OOH) during the reaction. Conversely, some computational^[^ ^84d^ ^]^ and experimental ^[^ ^83a^ ^]^ studies suggested that faster OER kinetics occur directly at Fe sites. For example, the high‐energy resolution fluorescence‐detected X‐ray absorption spectroscopy revealed that Fe^3+^ at the O_h_ sites in Ni_1−_ *_x_* Fe*_x_* OOH has short Fe─O bond distances, which lead to near‐optimal adsorption energies for OER intermediates and low overpotentials at the Fe.^[^ ^84d^ ^]^ Another study revealed that FeOOH exhibits higher intrinsic OER activity but is electrically insulating and prone to dissolution under OER conditions.^[^ ^34^, ^99^ ^]^ CoOOH is a conductive and stable host matrix for Fe‐based active sites in Co_1−_ *_x_* Fe*_x_* (OOH). This is supported by voltammetry studies, which revealed the dependence of Co^2^⁺/Co^3+^ redox potential on the Fe content, suggesting significant electronic coupling between Fe and Co (Figure 4f).^[^ ^99^ ^]^ Additionally, others claimed that the high OER activity of Co‐ and Ni‐based electrocatalysts originates from the synergistic interaction between Fe and Ni/Co. For example, Xiao et al. utilized quantum mechanics methods to trace the formation of both Fe^4+^ and Ni^4+^ during polarization. They established that high‐spin d^4^ Fe^4+^ stabilizes the oxyl radicals that are generated by low‐spin d^6^ Ni^4+^, facilitating O─O bond formation.^[^ ^100^ ^]^

Furthermore, the redox peak position highly depends on the ratio of cations, which alters the OER activity accordingly. For instance, increasing the Co content in Ni*_x_* Co*_y_* (OH)*_z_* results in a negative shift in the anodic redox potential (Figure 4g).^[^ ^101^ ^]^ One might assume that shifting redox peaks to lower potentials indicates activation toward OER, but optimal activity is achieved at a moderate Co content.^[^ ^101^ ^]^ In Ni─Fe oxides or hydroxides, the Ni^2+^/Ni^3+^ redox peak shifts to higher potentials as Fe concentration increases from 0 to 35 at%, while OER activity improves continuously.^[^ ^86^ ^]^ These results imply that shifts in redox peak positions cannot solely predict OER activity. Some studies also point out that cation deficiencies^[^ ^102^ ^]^ and/or oxygen vacancies^[^ ^103^ ^]^ can shift redox potentials, leading to earlier anodic oxidation and higher OER activity. Additionally, variations in the shape, position, and intensity of redox peaks could reflect that the electrocatalyst undergoes reversible or irreversible changes, which may affect its stability and activity.^[^ ^31^, ^104^ ^]^ For example, the intensity decrease and positive shift of the redox peaks of Ni(OH)_2_ during continuous CV cycles have been associated with degradation processes due to irreversible phase change under OER conditions.^[^ ^104^ ^]^ Other studies^[^ ^97^, ^105^ ^]^ have noted similar structural changes and redox potential shifts, which suggest that analyzing redox peak behavior is essential for assessing the activity and stability of OER electrocatalysts. The potential of redox peak positions is likely associated with the binding energies of key intermediates during OER. More research is required in forthcoming studies to clarify the chemical nature of redox‐active sites and surface transformation. This will offer new opportunities to improve OER activity by adjusting redox behaviors.^[^ ^84^, ^99^, ^100^, ^106^ ^]^

### 3.3 Surface Amorphization and Transformation

Designing spinel or (oxy)hydroxide electrocatalysts for water oxidation requires a comprehensive understanding of the thermodynamic and kinetic properties at the electrode/electrolyte interface.^[^ ^108^, ^119^ ^]^ The electrocatalyst surface's geometrical, chemical, and electronic nature dominates the activity at the onset of OER. However, the electrocatalyst surfaces change dynamically due to successive chemisorption and deprotonation processes at high anodic potentials.^[^ ^120^ ^]^ Surface reconstruction, transformation, cation dissolution, and (re)deposition occur on electrocatalyst surfaces. These elementary processes can result in either activation or deactivation of the electrocatalysts. Therefore, it is essential to thoroughly understand the origins and dynamics of the elementary processes and their impact on the activity and stability to aid the design of efficient OER electrocatalysts.^[^ ^97b^ ^]^

**Figure 5:**
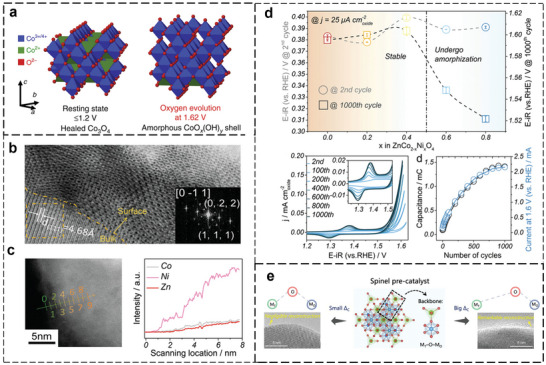
a) At potentials below Co redox features, Co_3_O_4_ is in a healed state at which defects in the near surface are oxidized. At elevated O_2_ evolution, the CoO*_x_* (OH)*_y_* grows into the crystalline Co_3_O_4_ core leading to a reversible amorphization of a sub‐nanometer shell. This amorphous CoO*_x_* (OH)*_y_* shell consists of di‐µ‐oxo‐bridged Co^3+/4+^ ions with arbitrary site occupancy in the ideal cubic close‐packed O^2−^ lattice. Reproduced (Adapted) with permission.^[^ ^122^ ^]^ Copyright 2015, Springer Nature. b) TEM image of ZnCo_1.2_Ni_0.8_O_4_ after a thousand cycles of OER. The inset at the bottom right shows the FFT pattern for the bulk which can be referred to *Fd*3*m* space group. c) Scanning transmission electron microscopy–energy dispersive X‐ray spectroscopy (STEM–EDS) image and analysis of cycled ZnCo_1.2_Ni_0.8_O_4_. d) The top figure shows the *iR*‐corrected potential (in RHE scale) of the 1000th cycle in comparison to the 2nd cycle of ZnCo_2−_ *_x_* Ni*_x_* O_4_ (*x* = 0.0, 0.2, 0.4, 0.6, 0.8) at 25 µA cm^−2^ oxide. The bottom shows the evolutive CV curves for representative ZnCo_1.2_Ni_0.8_O_4_ from the 2nd to the 1000th cycle in 1 m KOH at 10 mV s^−1^ between 0.904 and 1.624 V (vs RHE). The insets show the redox peaks from 1.23 to 1.5 V with the evolution of the pseudocapacitive charge during cycling (0.904–1.624 V vs RHE) in 1 m KOH (left axis) and the OER activity at 1.6 V versus RHE (right axis) for ZnCo_1.2_Ni_0.8_O_4_. Reproduced (Adapted) with permission.^[^ ^110^ ^]^ Copyright 2019, John Wiley and Sons. e) Schematic of the structure–reconstruction relationship for spinel precatalyst during water oxidation. Reproduced (Adapted) with permission.^[^ ^97b^ ^]^ Copyright 2023, Springer Nature.

One of the most frequently observed phenomena occurring during OER is the surface amorphization of the electrocatalysts.^[^ ^120^ ^]^ Surface amorphization, often detected by transmission electron microscopy (TEM), is when the electrocatalyst surfaces lose the pristine crystal structures' medium‐ or long‐range atom arrangements. Another type of “amorphous” surface layer reported in the literature is X‐ray amorphous,^[^ ^121^ ^]^ which is mainly driven by surface transformation to nanosized active surface species that broadens the X‐ray spectra. Bergmann et al.^[^ ^122^ ^]^ observed by advanced in situ X‐ray techniques that the sub‐nanometer thick surfaces of Co_3_O_4_ spinel transform into X‐ray amorphous CoO*_x_* (OH)*_y_* composed of di‐µ‐oxo‐bridged Co^3+/4+^ at OER potentials (1.62 V vs RHE), below which such catalytically active X‐ray amorphous layer reverts to the spinel structure (**Figure** **5**a). The tetrahedrally coordinated, mono‐µ‐oxo‐bridged Co^2+^ ions were found to be reversibly converted into octahedrally coordinated, di‐µ‐O(H)‐bridged Co^3+/4+^ ions.^[^ ^122^ ^]^ In addition to the surface transformation, metal leaching also induces surface amorphization. For instance, Duan et al. observed that activated ZnCo_2−_ *_x_* Ni*_x_* O_4_ spinel surfaces become amorphous due to the leaching of Zn and Co (Figure 5b,c).^[^ ^110^ ^]^ The continuous Zn and Co dissolution exposes the Ni sites on the surfaces, forming OER‐active NiOOH species and contributing to the activation of ZnCo_2−_ *_x_* Ni*_x_* O_4_ (Figure 5d). Another study claimed that Zn^2+^ leaching in Zn*_x_* Ni_1−_ *_x_* Co_2_O_4_ spinel results in the formation of Zn vacancy (V_Zn_)─O─Co surface active sites responsible for the OER activity.^[^ ^105b^ ^]^ Essentially, such defective surfaces, induced by cation dissolution, likely promote the formation of active oxyhydroxides that enhance the OER activity, as consistently observed in various Co‐based spinels such as CoCr_2_O_4_,^[^ ^92a^ ^]^ CoAl*_x_* Fe_1−_ *_x_* O_4_,^[^ ^97c^ ^]^ LiNiO_2_^[^ ^112^ ^]^ in alkaline media. However, the cation leaching might not be the direct driving force for the surface transformation. Instead, the oxygen vacancies created by cation dissolution (to maintain the charge neutrality of the spinels) potentially promote the surface transformation to active (oxy)hydroxides during OER. This is evidenced by a recent study^[^ ^103a^ ^]^ that oxygen‐vacancy‐rich Co_3_O_4_ (termed V_O_─Co_3_O_4_) exhibited a faster oxidation process as opposed to the pure Co_3_O_4_, whereby the authors concluded that the oxygen vacancies initiated the surface reconstruction into CoOOH species before the OER process, enhancing the OER activity.

To better understand and exploit such surface reconstruction to enhance the OER activity of spinels, Xu and co‐workers performed DFT calculations and pre‐ and postcatalytic electrochemical analysis on the Li*_x_* Co_3−_ *_x_* O_4_ spinel as a model system.^[^ ^97b^ ^]^ They observed a linear relationship between covalency polarity, defined as the difference in the bond strengths between tetrahedrally and octahedrally coordinated M─O bonds, and the amorphous surface layer's thickness that indicates the extent of surface reconstruction. This result infers that the spinels with a stronger M_Oh_─O covalency were thought to experience more pronounced surface reconstruction with enhanced OER activity (Figure 5e). Given that the cations in the oxyhydroxides remain in O_h_ coordination, stronger metal–oxygen covalency in octahedrally coordinated sites in spinels aids the surface reconstruction. Thus, metal–oxygen (M─O) covalency polarity and M_Oh_─O covalency in spinels were proposed to play a decisive role in surface reconstruction.^[^ ^97b^ ^]^

Notably, surface transformation may not always lead to activity enhancement, as the intrinsic activity of some electrocatalyst materials can be higher than that after surface reconstruction and transformation. For instance, inactive CoO_2_ nanodomains were found to be grown on the surface of Co_2_FeO_4_ nanoparticles after long OER cycling (1000 cycles), leading to electrocatalyst deactivation.^[^ ^31^ ^]^ Additionally, although most studies reported that the activated OER electrocatalyst surfaces are amorphous,^[^ ^123^ ^]^ the surface amorphization does not always indicate the activation of electrocatalysts, as revealed in a recent study that inactive amorphous La(OH)_3_ layer was formed on the LaCoO_3_ nanoparticle surfaces at OER potentials.^[^ ^124^ ^]^ Additionally, many studies in the literature speculated that the surface amorphization is due to the formation of active oxyhydroxides without providing any experimental verification, possibly because the characterization of such thin layers (approximately few nanometers) with low or no crystallinity remains challenging.^[^ ^125^ ^]^ In this regard, the term surface amorphization shall not be overused as the sole explanation for the activity improvement of OER electrocatalysts. Identifying the chemical nature of the in situ formed surface species within the amorphous layer is essential for understanding their precise role in enhancing electrocatalyst performance.

Additionally, surface reconstruction and transformation might lead to changes in the electrical conductivity and electrochemical capacitance of the OER electrocatalysts. For example, active species like defective Co‐ and Ni‐based (oxy)hydroxides are formed on the surfaces of Co‐ or Ni‐based spinels.^[^ ^126^ ^]^ The electrochemical capacitance of these oxyhydroxides is lower than the spinel counterparts. In this regard, it is unreasonable to evaluate the changes in electrochemical active surface areas by directly comparing ECSAs after OER since *C*_s_ changes after surface transformation from spinel oxides to oxyhydroxides. Also, in situ formed (oxy)hydroxides can increase or decrease the electrical conductivity of the spinel electrocatalysts, depending on the types of (oxy)hydroxides, which in turn increase or decrease the OER current densities. Such changes in electrical conductivity also affect measured Tafel slopes.^[^ ^123b^ ^]^ Thus, although the in situ transformed (oxy)hydroxide layer improves the OER activity, it is difficult to conclude that the intrinsic activity of this surface reconstructed layer is better than pristine spinels toward OER as electrochemical capacitance and electrical conductivity also affect the OER current densities, overpotentials, Tafel slopes, and ECSAs. Additionally, such a gel‐like (oxy)hydroxide layer might promote hydroxylation, incorporation, or intercalation of electrolytes accessible to more active sites, improving OER activity.^[^ ^105^, ^123^ ^]^

### 3.4 Cation Dissolution

The extent of metal dissolution potentially influences surface reconstruction and transformation. Understanding why and which cations dissolve, when dissolution occurs, and how much cations dissolve is essential for understanding the role of cation dissolution in surface reconstruction and transformation. Such experimental evidence is also critical for establishing the structure–activity–stability relationships.

**Figure 6:**
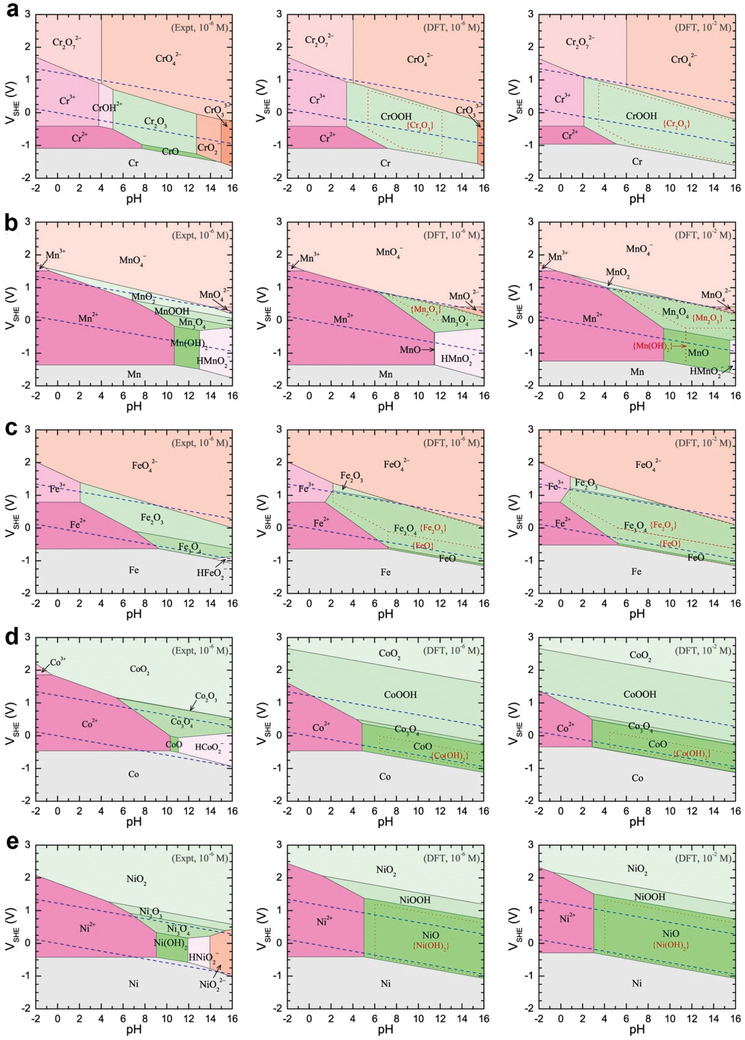
Experimental (*E*_xpt_) Pourbaix diagrams and DFT Pourbaix diagrams for a) Cr, b) Mn, c) Fe, d) Co, and e) Ni with different pH values. The two inclined parallel blue dashed lines indicate the electrode potential for the oxidation (upper) and reduction (lower) of water. Reproduced (Adapted) with permission.^[^ ^132^ ^]^ Copyright 2019, Springer Nature.

The types of metal cations, surface environment (e.g., applied potentials, electrolyte ions, and pH values), and the local electronic structure determine the metal dissolution.^[^ ^92^, ^127^ ^]^ Recent developments of scanning flow cell coupled to an inductively coupled plasma mass spectrometer (SFC–ICP–MS),^[^ ^128^ ^]^ have enabled researchers to elucidate the dissolution processes at varying potentials during the electrocatalytic reactions.^[^ ^129^ ^]^ For instance, online ICP–MS measurements of Co_3_O_4_ spinel oxides revealed that Co transient dissolution occurred at a constant potential of 0.5 V versus RHE.^[^ ^130^ ^]^ The amounts of dissolved Co decreased to a negligible quantity between 0.5 and 1.5 V versus RHE before the onset of OER but increased again upon cycling at 0.5 V versus RHE, possibly due to the reduction of Co_3_O_4_ to Co(OH)_2_.^[^ ^130^ ^]^ In addition to the transient dissolution, both anodic and cathodic dissolution occur upon OER cycling, which is associated with transformation. For example, Mn dissolution in Mn oxides during OER is potentially caused by the formation of thermodynamically favored soluble MnO_4_^2−^ or MnO_4_^−^ species or from constant surface state changes OER cycling.^[^ ^131^ ^]^ In this regard, Pourbaix diagrams^[^ ^132^ ^]^ guide to predict the thermodynamic equilibrium states at varying pH and potentials, as summarized for Cr, Mn, Fe, Co, and Ni in **Figure** **6**. We can see from Figure 6 that Fe, Mn, and Cr are likely to transform to high oxidation state soluble species such as FeO_4_^2−^, MnO_4_^−^, and CrO_4_^2−^ at high anodic potentials in alkaline media or the cations directly dissolve in an acid environment.^[^ ^132^, ^133^ ^]^

**Figure 7:**
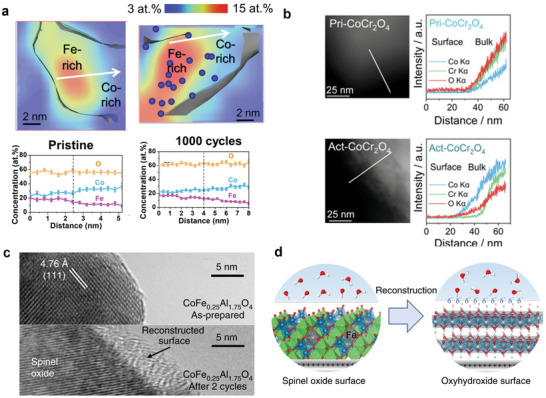
a) 3D‐atom probe tomography (APT) reconstruction of Co_2_FeO_4_ in the pristine state and after 1000 cycles of CV showing the nanoparticles embedded in Ni matrix, and the corresponding 3D atom maps of segregated Co_2_FeO_4_ nanoparticles in the pristine state and after 1000 cycles, selected from dashed line region. Reproduced (Adapted) with permission.^[^ ^31^ ^]^ Copyright 2022, Springer Nature. b) HAADF image of Pri‐CoCr_2_O_4_ and STEM–EDS image and analysis for Pri‐CoCr_2_O_4_, bottom HAADF image of Act‐CoCr_2_O_4_ and STEM–EDS image and analysis for Act‐CoCr_2_O_4_. Reproduced (Adapted) with permission.^[^ ^93a^ ^]^ Copyright 2021, John Wiley and Sons. c) HRTEM images showing the surface regions for as prepared and after two cycles of CoFe_0.25_Al_1.75_O_4_ sample. d) Schematic illustration of reconstruction process from spinel CoFe_0.25_Al_1.75_O_4_ into oxyhydroxide with activated negatively charged oxygen ligand. Reproduced (Adapted) with permission.^[^ ^97c^ ^]^ Copyright 2019, Springer Nature.

The dissolution of metal cations in spinels is generally thought to deteriorate long‐term stability due to material depletion. For instance, Co_2_FeO_4_ exhibits superior activity at the onset of OER due to the formation of (Co, Fe)OOH.^[^ ^31^ ^]^ As the reaction proceeds, substantial amounts of Fe dissolve from the Co_2_FeO_4_ nanoparticle surfaces, possibly in the form of FeO_4_^2−^, leading to a pronounced OER activity drop (see 2D and 1D concentration profiles of Co_2_FeO_4_ nanoparticles before and after 1000 cycles of OER in **Figure** **7**a).^[^ ^31^ ^]^ On the other hand, cation dissolution can also promote OER activity due to increased surface areas and exposure of more active metal sites. For instance, Driess and co‐workers^[^ ^117^ ^]^ reported that ZnCo_2_O_4_ exhibited a superior OER activity compared to Co_3_O_4_ because the redox‐inactive tetrahedral‐coordinated Zn^2+^ dissolves, which creates additional cation vacancies for hydroxylation and exposes a higher amount of accessible Co^3+^_Oh_ sites that are thought to be active for OER.^[^ ^117^ ^]^ Similarly, Cr, Mn, Fe, and V with octahedral coordination in spinels are speculated to leach into alkaline electrolytes.^[^ ^134^ ^]^ For instance, Cr dissolves in CoCr_2_O_4_ at high potentials (OER potentials), as revealed by the scanning transmission electron microscopy (STEM) image in Figure 7b.^[^ ^92a^ ^]^ The high OER potential triggers the anodic oxidation of Cr ions from Cr^4+^ into Cr^6+^ (in the form of CrO_4_^2−^), creating more vacancies and defects that promote surface transformation to active (oxy)hydroxides for OER. Notably, such transformation and dissolution of high oxidation state soluble species can be intervened by tuning its ratio with other 3d transition‐metal cations in spinels, possibly due to changes in the number of the octahedrally coordinated sites. For instance, MnO_4_^−^ is responsible for the activity loss of Mn spinel oxides after only the first few cycles.^[^ ^135^ ^]^ However, adding Co in Mn spinel oxides shows nearly unchanged OER current densities with negligible changes in the Tafel slope of (Co_0.7_Mn_0.3_)O*_x_* after long durations of OER.^[^ ^135b^ ^]^ The presence of more Co sites could impede the Mn dissolution. Additionally, our recent work elucidated that dynamic Mn dissolution and redeposition might occur in the CoMnOOH layer grown on Co_2_MnO_4_ during OER cycling, extending the stability toward OER.^[^ ^29^ ^]^ Therefore, cation dissolution is not always detrimental to the OER electrocatalyst activity and stability since some cation dissolution increases the exposure of active sites to electrolytes and promotes surface transformation that improves the OER performance.

Moreover, the relative location of the O 2p‐band center and Fermi level might also influence the cation leaching.^[^ ^97c^ ^]^ Specifically, the upshift of the O 2p states toward *E*_F_ possibly leads to the lattice oxygen mechanism for OER, as confirmed by many theoretical studies for A*_x_* B*_y_* O*_z_* oxides (perovskites and spinels), wherein cation dissolution is also facilitated that induces surface amorphization.^[^ ^97c^ ^]^ For instance, in Fe‐substituted CoFe*_x_* Al_2−_ *_x_* O_4_, adding Fe uplifted the O 2p center near the Fermi level, inducing Al^3+^ leaching at the onset of OER. After the initial Al^3+^ dissolution, the energy level of O 2p decreases, allowing for surface reconstruction (Figure 7c,d) and oxidation via lattice oxygen, leading to high activity and stability.^[^ ^97c^ ^]^

### 3.5 (Re)Deposition on the Ni‐ and Co‐Based Electrocatalysts

**Figure 8:**
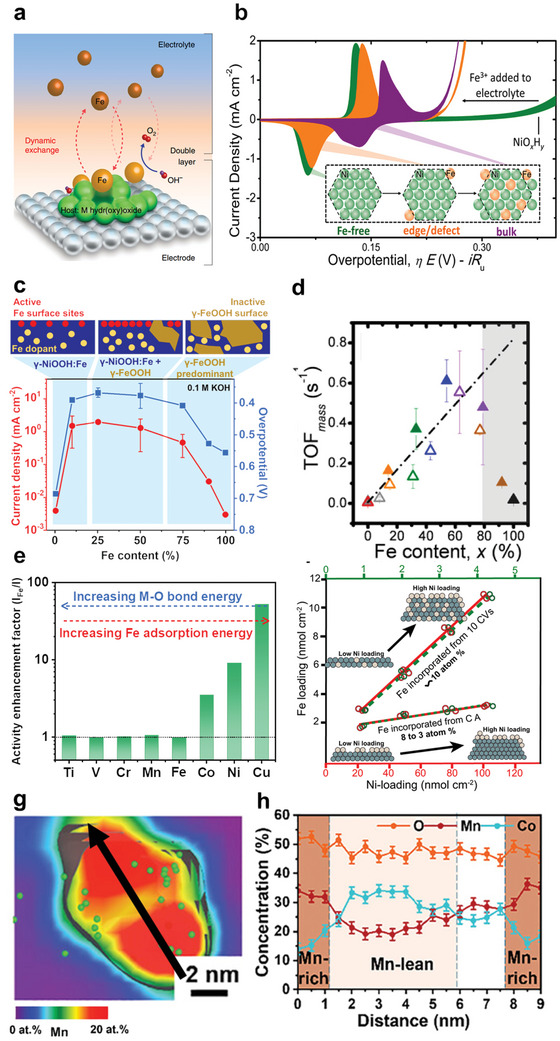
a) Schematic diagram of the dynamic Fe dissolution and redeposition on the surface of metal hydr(oxy)oxides. Reproduced (Adapted) with permission.^[^ ^136^ ^]^ Copyright 2020, Springer Nature. b) CV curves of NiO*_x_* H*_y_* cycled (10 mV s^−1^) in Fe‐free 1 m KOH (green line), and the 11th cycle (orange) and 24th cycle (purple) in 1 mm Fe spiking KOH. The inset depicts a possible schematic of Fe incorporation into the edge and bulk site of NiO*_x_* H*_y_*. Reproduced (Adapted) with permission.^[^ ^83a^ ^]^ Copyright 2017, American Chemical Society. c) OER activity (current densities at *η* = 0.3 V and overpotential@10 mA cm^−2^) of mixed Ni─Fe catalysts as a function of Fe content in 0.1 m KOH. Top: a schematic illustrating the influence of Fe content on the competing formation of highly active Fe sites in γ‐NiOOH and of phase‐separated low‐activity γ‐FeOOH. Reproduced with permission^[^^]^ Copyright 2015, American Chemical Society. d) The turnover frequency (TOF_mass_) of different compositions of Co_1−_ *_x_* Fe*_x_* (OOH) film during steady‐state polarization at *η* = 350 mV after 1 min (closed symbols) and 120 min (open symbols). Reproduced (Adapted) with permission.^[^ ^99^ ^]^ Copyright 2015, American Chemical Society. e) The activity enhancement of 3d transition‐metal (oxy)hydroxides by Fe incorporation along with the increasing Fe adsorption energy, which is inverse correlated with the M─O bond energy. Reproduced (Adapted) with permission.^[^ ^136^ ^]^ Copyright 2020, Springer Nature. f) The amount of Fe incorporated into NiO*_x_* H*_y_* film during CA and CA plus 10 CV cycles versus the total Ni mass loading and film capacitance. Reproduced (Adapted) with permission.^[^ ^140^ ^]^ Copyright 2023, Springer Nature. g,h) APT 2D compositional map of Mn in CoMn_2_O_4_ nanoparticles after 500 CV cycles, and the responding 1D concentration profiles along the black arrows. Reproduced (Adapted) with permission.^[^ ^29^ ^]^ Copyright 2024, John Wiley and Sons.

The most commonly reported (re)deposition process is that Fe in the electrolytes deposits on the surfaces of NiO*_x_* H*_y_* and CoO*_x_* H*_y_* during OER cycling, whereby their OER activity is enhanced significantly.^[^ ^136^ ^]^ Essentially, Fe also dissolves from the electrocatalysts, but the intense adsorption energy between Fe ions in the electrolyte and NiO*_x_* H*_y_* ^[^ ^83a^ ^]^ or CoO*_x_* H*_y_* ^[^ ^99^ ^]^ substrates enables a faster rate of Fe redeposition than that of the Fe dissolution, providing dynamically stable Fe active sites (**Figure** **8**a).^[^ ^136^ ^]^ The processes of Fe deposition and incorporation on, e.g., NiO*_x_* H*_y_*, could be inferred from the redox peaks in the CV curves (Figure 8b). Specifically, Fe first deposits on easily accessible edge/defect sites of the NiO*_x_* H*_y_* at initial OER cycles, forming Ni─O─Fe species that induce one pair of redox peaks in the CV curve (Figure 8b, orange curve). As OER proceeds, Fe ions are gradually incorporated into NiO*_x_* H*_y_* bulk, which leads to two pairs of redox peaks reflecting an uneven distribution of Fe atoms in the bulk and on the edges of the NiO*_x_* H*_y_* (Figure 8b, purple curve).^[^ ^83a^ ^]^ Notably, Fe exists as impurities in the electrolyte, which can significantly increase the OER activity of (oxy)hydroxides or spinels.^[^ ^137^ ^]^ This makes the findings of most OER electrocatalyst studies elusive, as it is difficult to decouple the role of Fe (in the unpurified electrolyte) from the intrinsic OER activity of the electrocatalysts. Thus, purification of the electrolytes should be performed before electrochemical measurements of OER electrocatalysts.^[^ ^99^, ^137^ ^]^

After establishing the electrolyte purification protocols, Friebel and co‐workers demonstrated that the OER current density of Ni oxyhydroxide film can be enhanced by up to 500‐fold higher after adding Fe to NiOOH (Ni_0.75_Fe_0.25_OOH) than the pure NiOOH films (Figure 8c).^[^ ^84d^ ^]^ The presence of Fe in Ni oxyhydroxides enhanced its electrical conductivity, contributing to the improvement of OER activity. In addition, Fe incorporation in the Ni (oxy)hydroxides leads to the formation of Ni─Fe LDH, i.e., Ni(II)_1−_ *_x_* Fe(III)*_x_* (OH)_2_(SO_4_)*_x_*_/2_(H_2_O)*_y_*, where Fe exhibits more optimal adsorption energies of OER intermediates, thereby enhancing OER activity.^[^ ^84d^ ^]^ Previous DFT simulation work also demonstrated that Fe^3+^ in γ‐NiOOH resulted in a significantly lower overpotential than Ni^3+^.^[^ ^84d^ ^]^ Similarly, Fe deposition and incorporation can activate Co (oxy)hydroxides.^[^ ^138^ ^]^ Boettcher and co‐workers compared the intrinsic OER activity of different compositions of Co_1−_ *_x_* Fe*_x_* (OOH) and found that Co_0.54_Fe_0.46_(OOH) exhibited a superior OER activity with the highest TOF, which is ≈100‐fold higher than that of pure CoOOH film (Figure 8d).^[^ ^99^ ^]^ However, the activation effect of Fe on Co oxyhydroxides is slower and weaker than that on Ni oxyhydroxides since adding 8% of Fe in CoOOH requires 2 h of OER cycling to attain approximately tenfold higher activity. By contrast, adding 5% of Fe in NiOOH only requires ≈10 min, yielding ≈100‐fold higher OER activity than its pristine state.

Despite these successful studies, the exact role of (re)deposited Fe in this improvement of Ni‐ and Co‐based (oxy)hydroxides remains elusive. Markovic and co‐workers^[^ ^99^ ^]^ proposed that establishing dynamically stable Fe sites through balancing the rates of Fe dissolution and redeposition on the (oxy)hydroxides is key to retaining the high OER activity and stability. The Fe adsorption energy to the (oxy)hydroxides substrates was proposed to serve as the activity descriptor of 3d transition‐metal (oxy)hydroxides in the alkaline media (Figure 8e).^[^ ^99^ ^]^ However, it should be noted that Fe has a maximum solubility limit of ≈25% for Ni(OH)_2_. As the Fe content increases, a separate γ‐FeOOH phase with lower electrical conductivity is formed, which leads to decreased activity (Figure 8f).^[^ ^84d^ ^]^ Different electrochemical conditions also affect Fe redeposition and incorporation in Ni‐ and Co‐based electrocatalysts. For instance, the OER performance of NiFe hydroxides remained stable after 50 CV cycles, while they suffered from a rapid drop in activity after the CA test.^[^ ^139^ ^]^ The dissolved Fe is thought to be redeposited at the edge sites of the NiOOH during the CA measurements, leading to the preferential formation of inactive FeOOH phase segregated from the Ni─Fe hydroxide lattice, which is responsible for the activity degradation. In comparison, the cathodic sweep during the CV measurements facilitates uniform redeposition of dissolved Fe cations into the Ni hydroxide matrix, preventing the formation of the FeOOH phase and retaining the high OER activity.^[^ ^139^ ^]^

Additionally, the (re)deposition processes, such as deposition sites, remain unclear. Yeo and co‐workers performed in situ XAS on CoO*_x_* under the OER condition by adding 0.3 mm Fe^3+^ in the electrolyte. The deposited Fe on CoO*_x_* was found to exhibit a lower coordination number, suggesting that the deposited Fe is likely surrounded by more oxygen vacancies that increase the electrical conductivity and are more accessible to OH^−^.^[^ ^141^ ^]^ Essentially, Fe (re)deposition and incorporation occurs most likely along with surface transformation, which makes it difficult to examine the Fe (re)deposition sites and processes. For instance, NiMoO_4_·*n*H_2_O was found to be activated during anodic polarization in Fe‐containing KOH.^[^ ^142^ ^]^ Fe deposition and incorporation took place during the formation of the NiOOH surface layer induced by Mo leaching. The resulting NiMoO_4_·*n*H_2_O@Fe─NiOOH electrocatalyst exhibited high OER activity with an overpotential of 227 mV at 10 mA cm^−2^. Also, Ni*_x_* Co*_y_* O_4_ (*x*/*y* = 1/4) could be activated by the Fe impurities in KOH electrolyte.^[^ ^143^ ^]^ During activation, the valence and chemical state of Co remains unchanged.^[^ ^122^ ^]^ By contrast, the Ni(OH)_2_ content increases, inducing the formation of Ni_1−_ *_x_* Fe*_x_* OOH species, which is responsible for the activity improvement.^[^ ^143^ ^]^

One of the key obstacles to better understanding the deposition process is that suitable characterization methods are significantly lacking. In situ or operando XAS,^[^ ^144^ ^]^ Raman spectroscopy,^[^ ^145^ ^]^ TEM,^[^ ^146^ ^]^ scanning transmission X‐ray microscopy (STXM),^[^ ^116^ ^]^ wide‐angle X‐ray scattering (WAXS),^[^ ^147^ ^]^ and infrared (IR) spectroscopy,^[^ ^148^ ^]^ can be powerful means to reveal active species or intermediates. However, characterizing cation dissolution and redeposition, such as dissolution depth profile from the electrocatalyst surface, the amounts and location of redeposited sites coupled with surface transformation remain challenging for most in situ or operando techniques. This requires a method that can provide elemental or composition analysis with sub‐nanometer spatial resolution, as the dissolved/redeposited regions can be within a few nanometers thick. High‐end characterization techniques, such as STEM/energy dispersive X‐ray spectroscopy (EDS), provide important insights into surface structure and composition. However, the inherently few nanometers scale of dissolution depth or surface reconstructed layer potentially renders them difficult to analyze in the 2D projected image or analysis. In this regard, our group developed multimodal approaches that combine atom probe tomography (APT), X‐ray‐based spectroscopies, TEM with online ICP–MS and electrochemical measurements to resolve surface structural and compositional changes of OER electrocatalysts.^[29,31,109,115,,124,149]^ For instance, our recent work unveiled that Mn dissolved and redeposited in CoMn_2_O_4_ upon OER cycling in alkaline media (Figure 8g,h). Such dynamic dissolution and redeposition process extends the stability of the Co─Mn spinels toward OER.^[^ ^29^ ^]^

Besides Fe (re)deposition, other cations have also been found to activate the OER activity of Ni‐ and Co‐based catalysts. Corrigan and Bendert studied the effect of 13 coprecipitated metals with an atomic content of ≈10% on the OER activity of Ni hydroxide.^[^ ^150^ ^]^ Among them, Ce, Fe, and La were shown to significantly lower the OER overpotential by up to 140 mV, while Pb, Cd, Zn, and Cr were found to decrease the OER activity of Ni hydroxide. The other cations, such as Co, Cu, Ag, Mn, Mg, and Y, showed minimal effect on the OER. Also, Tüysüz and co‐workers investigated the effect of the first‐row transition‐metal cations (Sc, Cr, Mn, Ni, Cu, and Zn) in cobalt ink solutions on the electrochemical transformation of Co(NO_3_)_3_^[^ ^138^ ^]^ and found out that most transition metals, especially Fe and Cu, promoted the formation of active CoOOH species or improved the conductivity, leading to increased current density and decreased overpotential. Qu and co‐workers deposited Ce^3+^ and PO_4_^3−^ on NiCo_2_O_4_ nanowires using the chemical deposition method.^[^ ^151^ ^]^ The good oxophilicity of Ce^3+^ could promote the adsorption of oxygen‐containing species and facilitate the formation and release of molecular oxygen, while PO_4_^3−^ can accelerate the deprotonation process of M─OOH to form M─OO*. A combination of Ce^3+^ and PO_4_^3−^ leads to the enhanced activity of NiCo_2_O_4_.^[^ ^151^ ^]^

### 3.6 Facet Effect in Spinel, (Oxy)Hydroxides, and LDH on OER Activity

**Figure 9:**
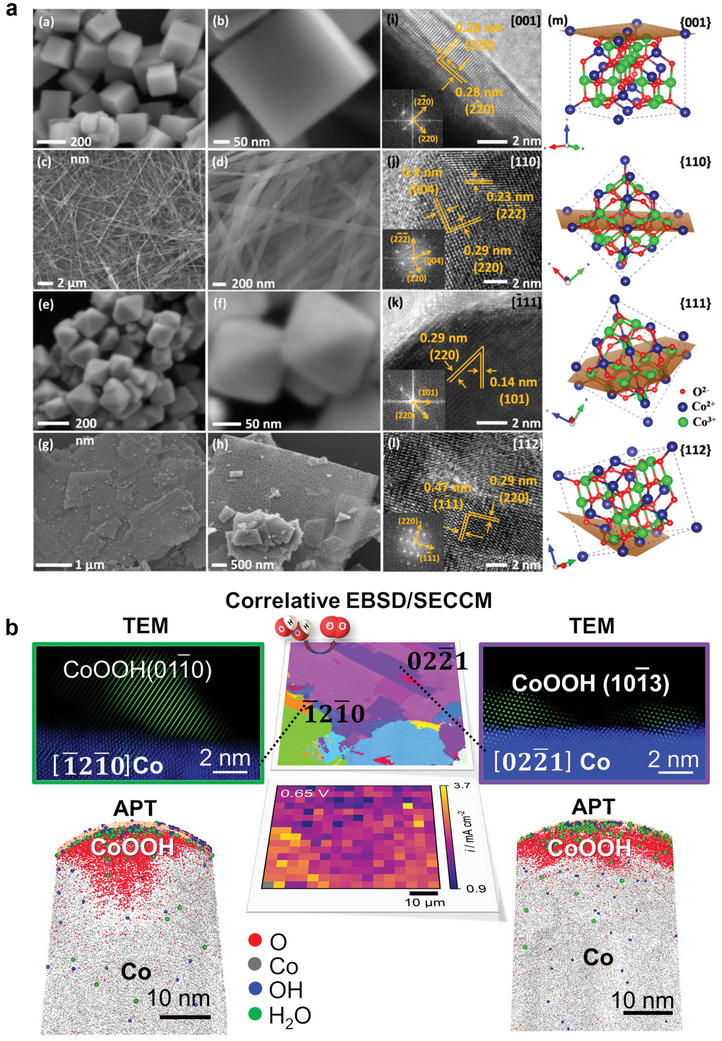
a) SEM and HRTEM images and the corresponding FFT patterns of the Co_3_O_4_ nanocube, Co_3_O_4_ nanobelt, Co_3_O_4_ nano‐octahedron, and Co_3_O_4_ nanosheet with the surface atomic configuration of Co_3_O_4_ in (001), (110), (111), and (112) facets. Reproduced (Adapted) with permission.^[^ ^154^ ^]^ Copyright 2017, American Chemical Society. b) Correlative scanning electrochemical cell microscopy (SECCM) electron backscatter diffraction (EBSD) maps of the 100‐cycle Co microelectrode showing the correlation of grain orientation and current normalized by estimated footprint size measured at 0.65 V versus RHE. Side‐view atom maps of [02$\bar{2}$1], and [$\bar{1}$2$\bar{1}$0]‐orientated Co after 100 CV cycles under OER conditions, showing in situ formation of (oxy)hydroxides. Reproduced (Adapted) with permission.^[^ ^109^ ^]^ Copyright 2023, John Wiley and Sons.

The intrinsic activity of the electrocatalysts is generally thought to be influenced by the specific crystal facets exposed on the surface since the atomic arrangement and coordination of the facets determine the number of active sites and thus binding energy with reaction intermediate.^[^ ^152^ ^]^ However, the impact of surface orientation on the OER activity and stability remains uncertain, as electrocatalyst's surfaces undergo substantial reconstruction and transformation. The formation of gel‐like, amorphous, or reconstructed layer likely destroys the atom arrangements on different orientations, potentially diminishing the influence of specific facets on OER activity. Despite this, several experimental and simulation studies demonstrate the correlation between specific crystal facets and the OER activity of spinels and (oxy)hydroxides. For instance, Tschulik and co‐workers showed that Co_3_O_4_ cubes with the (001) facets exposed on the surface exhibit higher OER activity compared to spheroids exposed by the (001) and (111) surface facets.^[^ ^153^ ^]^ Conversely, Liu et al. found that the electrocatalytic activity for overall water splitting decreased in the order of Co_3_O_4_ spinel dominated by (111) facets > nanosheets with exposed (112) facets > nanobelts enclosed by (110) facets > nanocubes bounded by (001) facets (**Figure** **9**a).^[^ ^154^ ^]^ Schulz and co‐workers^[^ ^155^ ^]^ also reported that the Co_3_O_4_ platelets with the predominant (222) facet exposure showed the highest OER performance, followed by cubic and spherical nanoparticles being the least active.^[^ ^155^ ^]^ Further DFT calculation work supported the observation and showed that the (111) facets have the highest surface energy and dangling bond density, which is responsible for the superior catalytic activity of Co_3_O_4_.^[^ ^154^ ^]^ By contrast, others claimed that the Co_3_O_4_(112) facet had better OER activity than (001) or (111) facets due to the presence of more octahedral coordinated Co^3+^_Oh_ sites that are responsible for the high OER activity.^[^ ^156^ ^]^

For (oxy)hydroxides and LDHs, facets were also found to affect the OER performance. For instance, the hierarchical structure of NiFe LDH nanosheets with more (012) edge plane exposure exhibited higher OER activity than those exposed by other facets.^[^ ^157^ ^]^ DFT simulations have confirmed that specific high‐index facet, e.g., (10$\bar{1}$4) of β‐CoOOH is the most active facet among (0001) and (01$\bar{1}$2) due to the presence of more Co^3+^ active sites.^[^ ^158^ ^]^ On the other hand, a recent DFT study^[^ ^159^ ^]^ claimed that the OER activity of β‐NiOOH is independent of crystallographic facet, since each low‐index facet, e.g., β‐NiOOH(0001), (10$\bar{1}$0), and ($\bar{1}$2$\bar{1}$1) has at least two types of active sites with similar overpotentials toward OER. Essentially, it is difficult to verify the DFT simulation results by experimental data since one must prepare single crystals to explore the facet effects of (oxy)hydroxides. Preparing single crystals with high‐indexed planes is challenging since hexagonal‐shaped (oxy)hydroxide platelets are generally enclosed by (0001) and (01$\bar{1}$0). Nevertheless, defect sites at the edge facets have been ascribed to the active sites in NiOOH,^[^ ^160^ ^]^ CoOOH,^[^ ^116^ ^]^ MnOOH,^[^ ^161^ ^]^ or FeOOH,^[^ ^162^ ^]^ contributing to a higher OER activity than those on the (0001) basal plane.

Despite the preliminary computation and experimental efforts, the facet effect of spinels and (oxy)hydroxides on the OER activity remains elusive. The conflicting experimental evidence of the facet effect possibly originates from surface reconstruction and transformation during OER. Also, the electrochemical performance varies spatially across a single electrocatalyst particle, as local surface defects can serve as active sites or promote the formation of active species.^[^ ^116^ ^]^ However, evaluating the activity of local active features is challenging for standard electrochemical measurements as they only provide integral electrochemical data over the entire electrode surface. To address this challenge, our group developed a multimodal characterization approach with a combination of scanning electrochemical cell microscopy (SECCM), electron backscatter diffraction (EBSD), TEM, and APT to establish the relationships between the OER activity with oxidation state, atomistic structure, elemental distribution, and composition of the Co (oxy)hydroxides that are formed on different facets on Co microelectrode (Figure 9b).^[^ ^109^, ^115^ ^]^ We unveiled that ≈6 nm β‐CoOOH(01$\bar{1}$0), grown on [$\bar{1}2\bar{1}$0]‐oriented Co, exhibits higher OER activity than ≈3 nm β‐CoOOH(10$\bar{1}$3) or ≈6 nm β‐CoOOH(0006) formed on [02$\bar{2}$1]‐ and [0001]‐oriented Co, respectively. This is possibly due to higher amounts of incorporated hydroxyl ions and more easily reducible Co^III^─O sites present in β‐CoOOH(01$\bar{1}$0) than those in the latter two oxyhydroxide facets.^[^ ^109^ ^]^ The formation of reversible active Co^III^─O sites is thought to be highly dependent on the surface facets of precatalysts.

### 3.7 Electrolyte Effect on the OER Activity

Electrolytes play a critical role in OER, as they provide necessary reactants, viz., water, protons, and/or hydroxyl groups, supply ions that act as the charge carriers, and serve as buffers to maintain a stable local pH on the electrocatalyst surface during OER.^[^ ^163^ ^]^ The electrolyte pH affects the OER reaction kinetics and the chemical nature of the electrocatalysts’ active sites.^[^ ^164^ ^]^ Specifically, the local pH influences the binding energies of reaction intermediates, such as OH*, O*, and OOH*, which alters the energy barriers for each reaction step, thus impacting the overall OER efficiency. Especially in alkaline media, OER proceeds via adsorption, deprotonation of hydroxide ions, and formation of oxygen species (e.g., oxyhydroxides) on the electrocatalyst surfaces. Interestingly, the dependence of OER activity on pH varies among different electrocatalysts. For instance, some electrocatalysts such as La_0.5_Sr_0.5_CoO_3−_ *_δ_*,^[^ ^165^ ^]^ ZnCoOOH^[^ ^166^ ^]^ exhibit pH‐dependent OER activity, while others like LaCoO_3_^[^ ^165^ ^]^ do not. This was explained by different OER mechanisms on these electrocatalysts.^[^ ^167^ ^]^ For the conventional adsorbate evolution mechanism, the OER process involves four concerted proton–electron transfer reaction steps, where the proton and the electron are transferred simultaneously. Given that the proton transfer is coupled with the electron transfer, the OER activity of, e.g., LaCoO_3_^[^ ^165^ ^]^ is thus independent of the pH. Conversely, the lattice‐oxygen‐mediated mechanism involves nonconcerted proton–electron transfer steps with proton transfer as the rate‐determining step. Variation in pH affects the availability of protons in the electrolyte,^[^ ^163^ ^]^ thereby influencing the reaction kinetics of, e.g., La_0.5_Sr_0.5_CoO_3−_ *_δ_*.^[^ ^165^ ^]^ Beyond kinetic effects, the electrolyte pH also influences the structural transformation of electrocatalysts during OER. For instance, Strasser and co‐workers observed that increasing the KOH concentration induced the contraction of the interlayer distance in NiFe LDH at 1.6 V versus RHE under OER conditions, which accelerated the structural transformation from α‐NiFe LDH into the catalytically active γ‐NiFe LDH phase.^[^ ^168^ ^]^ At the same time, higher pH shifts the redox peak of the Ni^2+^/Ni^3+/4+^ transition in Ni─Fe oxyhydroxide cathodically and enhances the peak intensity.^[^ ^168^, ^169^ ^]^

**Figure 10:**
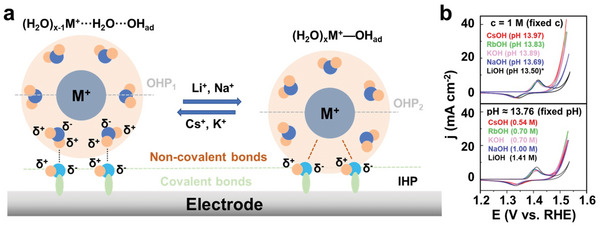
a) Schematic diagram of the generation of OH_ad_─M^+^(H_2_O)*_x_* clusters on the electrode surface. Reproduced (adapted) with permission.^[^ ^171^ ^]^ Copyright 2009, Springer Nature. b) The OER activity comparison of Ni_65_Fe_35_(OOH) in different alkaline electrolytes at fixed concentration and fixed pH value. Reproduced (Adapted) with permission.^[^ ^173^ ^]^ Copyright 2020, Springer Nature.

Besides pH, cations in alkaline electrolytes also play an important role in determining the OER performance. For example, in 0.1 m alkaline electrolytes, the OER activity of NiOOH follows the trend of Cs^+^ > K^+^ ≈ Na^+^ ≈ Li^+^.^[^ ^170^ ^]^ When Fe was added to the electrolyte, the OER activity in all electrolytes was enhanced significantly, but the trend shifted to Na^+^ ≈ K^+^ > Cs^+^ > Li^+^.^[^ ^170^ ^]^ Efforts have been devoted to investigating the effects of alkaline metal cations on the OER activity of (oxy)hydroxides. An in situ Raman study revealed that forming an active NiOOH phase with a slightly longer Ni─O bond length was promoted in CsOH, which is responsible for the higher OER current density.^[^ ^170^ ^]^ Additionally, Markovic and co‐workers proposed that OH_ad_─M^+^(H_2_O)*_x_* clusters form through noncovalent interactions between hydrated alkaline metal cations M^+^(H_2_O)*_x_* and adsorbed OH species (OH_ad_) on the Pt catalyst surface, as shown in **Figure** **10**a.^[^ ^171^ ^]^ Given that the Li^+^(H_2_O)*_x_* exhibits the strongest interaction energy with OH_ad_ among the alkali cations (Li^+^ > Na^+^ > K^+^ > Cs^+^), the highest coverage of these clusters can be achieved on the electrode surface in the LiOH electrolyte. However, these clusters are electrochemically inactive and block the active sites on the electrode surfaces.^[^ ^171^ ^]^ Groß and co‐workers further demonstrated that Li^+^ occupies a larger volume at the electrode surface due to its larger effective size (7.6 Å) than Cs^+^ (6.6 Å), leading to a lower OH^−^ concentration and inferior OER activity, based on the DFT calculation combined with microkinetic analysis, and a mean‐field submodel of the electrical double layer.^[^ ^172^ ^]^ Contrarily, some researchers proposed that the alkaline cations did not affect the intrinsic OER activity of Ni─Fe oxyhydroxide.^[^ ^173^ ^]^ Essentially, the basicity increases in the order of LiOH < NaOH < KOH < RbOH < CsOH due to increased amounts of dissociated OH^−^, which leads to a slight difference in pH values at the same concentration. When the pH values were adjusted to the same level, the OER activity of Ni_65_Fe_35_(OOH) was similar in different alkaline electrolytes.^[^ ^173^ ^]^ This suggests that the observed dependence of OER activity on alkaline cations is primarily due to differences in electrolyte pH (Figure 10b). Therefore, the effects of alkaline metal cations in the OER activity remain a subject of debate.

Notably, the ions in the electrolyte can intercalate into the electrocatalysts like LDHs or (oxy)hydroxides, affecting the activity and stability of the reaction intermediates during OER. Operando soft XAS studies have shown that when the NiFeO*_x_* catalyst layer contacts the electrolyte, Na^+^ (in NaOH) can irreversibly enter the catalyst at a low potential of 1.0 V versus RHE.^[^ ^174^ ^]^ Garcia et al. also obtained the OER activity trend of Cs^+^ > Na^+^ > K^+^ > Li^+^ for NiOOH.^[^ ^175^ ^]^ They revealed that cations intercalate into NiOOH, and the bigger Cs^+^ cations can stabilize the intermediates more effectively than smaller alkaline cations, explaining the dependence of OER activity on the alkaline cation size.^[^ ^175^ ^]^ Similar to NiOOH, a recent study on CoOOH shows that the OER activity increases in the order of Cs^+^ > K^+^ > Na^+^ > Li^+^. Cs^+^ was found to increase Co oxidation state by enlarging the Co─O bonds, which in turn enhances the d‐band center of Co, optimizing the adsorption strength of oxygen intermediates and promoting OER kinetics.^[^ ^176^ ^]^ Additionally, the alkaline metal cations could bind the intermediates strongly and poison the electrocatalysts.^[^ ^177^ ^]^ For instance, the activity of both NiO*_x_* H*_y_* and Ni*_z_*_−1_Fe*_z_* O*_x_* H*_y_* in 0.1 m KOH decreased significantly after adding 1 mm Ca(OH)_2_ in the electrolyte.^[^ ^177^ ^]^ This is because Ca^2+^, with a large ionic radius and strong acidity, exhibits a high affinity to the O atoms and strongly binds to water and OER intermediates with the electrocatalyst's inner layers, preventing the intermediates from further participating in the OER process.

Thus, varying alkali metal cations affect the OER activity of electrocatalysts differently (see **Table** **3**). Electrocatalysts such as NiOOH, TiO_2_, IrO_2_, and Ba_0.5_Sr_0.5_Co_0.8_Fe_0.2_O_3−_ *_δ_* exhibit the lowest OER activity in LiOH,^[^ ^178^ ^]^ while CoOOH shows a different trend (NaOH > LiOH > KOH > CsOH).^[^ ^138^ ^]^ Ding et al. found that the OER activity of Co_3_O_4_, Fe(OH)_2_, and MnO*_x_* in different electrolytes follows the same activity trend: LiOH > KOH > NaOH.^[^ ^179^ ^]^ The superior OER activity in LiOH was attributed to its weakest interaction of O─H bond with Li^+^ based on the DFT simulation of O─H bond length after interacting with Li^+^(H_2_O)_3_, Na^+^(H_2_O)_3_, and K^+^(H_2_O)_4_ cations.^[^ ^179^ ^]^

**Table 3:** The activity order of various electrocatalysts in different alkaline electrolytes.

Catalyst	Reaction	Electrolyte condition	Activity order	Refs.
NiOOH	OER	0.1 m purified	Cs^+^ > K^+^ ≈ Na^+^ ≈ Li^+^	[170]
NiOOH	OER	0.1 m Fe‐saturated	Na^+^ ≈ K^+^ > Cs^+^ > Li^+^	
NiOOH	OER	0.1 m purified	Cs^+^ > Na^+^ > Li^+^	[172]
NiOOH	OER	0.1 m unpurified	Cs^+^ > Na^+^ > K^+^ > Li^+^	[175]
NiOOH	OER	0.1 m purified	Cs^+^ > Na^+^ > K^+^ > Li^+^	
Pt electrode	ORR, HOR	0.1 m	Cs^+^ > K^+^ > Na^+^ > Li^+^	[171]
IrO_2_, Ba_0.5_Sr_0.5_Co_0.8_Fe_0.2_O_3−_ *_δ_*	OER	0.1 m	K^+^ > Na^+^ > Li^+^	[178]
Ni_65_Fe_35_(OOH)	OER	1 m purified	Cs^+^ > Rb^+^ > K^+^ > Na^+^ > Li^+^	[173]
CoOOH	OER	1 m purified	Cs^+^ > K^+^ > Na^+^ > Li^+^	[176]
CoOOH	OER	1 m purified	Na^+^ > Li^+^ > K^+^ > Cs^+^	[138]
CoOOH	OER	1 m purified + Fe	Na^+^ > K^+^ > Cs^+^ > Li^+^	
Co_3_O_4_, Fe(OH)_2_, MnO*_x_*	OER	1 m	Li^+^ > K^+^ > Na^+^	[179]

## 4 Summary and Outlook

In summary, this review addresses critical issues with the electrochemical testing of 3d transition‐metal (oxy)hydroxides and spinel‐type oxides and how the elementary processes occurring during OER affect the electrocatalyst surface changes and their electrochemical performance. Despite the rapid development of OER electrocatalysts, some important aspects remain unclear and controversial, which require further investigation for advancing OER electrocatalyst design.

1. Multiple elementary processes induce the surface reconstruction and transformation of spinel oxides, while the origins of reconstruction and transformation remain elusive. Some speculated that the cation dissolution is the main driving force,^[^ ^92^, ^105^ ^]^ while others hypothesized that the ability of surface reconstruction and transformation is structure‐dependent, such as metal–oxygen covalency^[^ ^97b^ ^]^ and oxygen vacancy.^[^ ^103a^ ^]^ More importantly, neither surface transformation nor amorphization necessarily leads to an activation of electrocatalysts toward OER, which depends on the intrinsic activity of the precatalyst and its reconstructed counterpart. In this sense, establishing the relationships between dynamic changes in surface state (valence, structure, atom coordination, composition, and elemental distribution), activity, and stability is crucially important. Additionally, systematic studies to compare the surface state changes of various 3d transition‐metal spinels and correlate these changes with electrocatalytic performance are urgently needed in order to draw generalized knowledge, such as activation or deactivation mechanisms. This knowledge will be critical for theoreticians to propose new descriptors for activity and perform molecular dynamic simulations to predict the dynamic surface reconstruction and transformation during OER.
2. Although substantial and dynamic compositional and structural change occurs on the surfaces of 3d transition‐metal spinel‐type oxides and (oxy)hydroxides, facets are thought to determine the OER activity according to recent studies.^[^ ^109^, ^153^ ^]^ The facets of spinels and (oxy)hydroxides are believed to determine the morphology, thickness, hydroxylation, structure, and composition of reconstructed intermediates that determine the OER activity. Thus, a better understanding of how various spinels and (oxy)hydroxide facets facilitate the growth of active species is thought to be the next step in engineering facets for enhanced OER performance. With this knowledge, researchers can prepare particular surface facets or defects of pristine electrocatalysts that intentionally facilitate the formation of the most active species, promoting OER performance.
3. To evaluate electrocatalysts' OER activation/deactivation, one could measure ECSA before and after OER to estimate the changes in the active surface areas or sites.^[^ ^180^ ^]^ However, the specific capacitance and electrical conductivity could change due to the surface transformation of spinel‐type oxides and (oxy)hydroxides, which would lead to misleading interpretation as the specific capacitance 40 µF cm^−2^ is an inaccurate value for ECSA estimation. Although BET area can be measured precisely and correlated with the electrochemical nature of the catalysts, it cannot directly represent ECSA due to the different operating conditions between N_2_ adsorption/desorption and OER in alkaline electrolytes. Thus, estimating the accurate active surface area after OER is still challenging due to the surface structural transformation during the reaction. New experimental protocols or techniques, such as electrochemical AFM, need to be developed for measuring ECSA for spinels and (oxy)hydroxides.
4. The effect of different alkaline cations or anions in electrolytes on OER activity remains debatable. Some concluded that the alkaline cations affect the formation of active intermediates differently, while others speculated that alkaline cations do not directly affect OER activity since electrolyte pH changes even at the same concentration of different alkaline electrolytes. In this regard, the pH values must be adjusted to the same value when investigating the role of alkaline cations in OER activity. Additionally, cations can intercalate into the electrocatalysts, stabilizing the OER intermediates and altering the reaction kinetics. A better understanding of the interfacial water and the reconstruction within the electrical double layer is essential. The cation and anion distribution at the electrical double layer affects the cation redeposition, especially Fe, since Fe incorporation on Ni and Co electrocatalysts promotes the OER activity substantially. This can be achieved by employing in situ techniques to investigate the roles of the electrolytes on surface reconstruction and transformation to better understand the electrolyte effects on the OER performance. In addition, developing computational methods to calculate the relative adsorption energies of reaction intermediates to model the orientation of water molecules at the electrode–electrolyte interface is crucially important.
5. New methods that can reveal surface state, including oxidation state, composition, structure, atom coordination, and elemental distribution, are required to be established to better characterize the changes of electrocatalyst surfaces during OER. In situ or operando techniques such as XAS,^[^ ^144^ ^]^ X‐ay photoelectron spectroscopy (XPS),^[^ ^181^ ^]^ X‐ray diffraction (XRD),^[^ ^182^ ^]^ Raman,^[^ ^14^, ^183^ ^]^ TEM,^[^ ^146^ ^]^ STXM,^[^ ^114^ ^]^ WAXS,^[^ ^147^ ^]^ IR spectroscopy,^[^ ^149^ ^]^ Ultraviolet–visible spectroscopy (UV–vis)^[^ ^184^ ^]^ and Electron energy loss spectroscopy (EELS)^[^ ^185^ ^]^ provide dynamic evolution of active sites, electronic structure, and intermediates of electrocatalyst surfaces during the reaction. In situ and operando techniques need to couple with other ex situ characterization techniques to provide a holistic view of the surface state since in situ or operando spectroscopies generally give one or two experimental details on the surfaces or bulk of electrocatalysts. Additionally, new nanoscopic techniques, such as APT with sub‐nanometer spatial resolution, are required to provide compositional and elemental details on the topmost few nanometers surface regions. Also, qualitative and quantitative experimental details of hydroxyl ions and water molecules can be particularly useful in estimating the degree of hydroxylation of the electrocatalyst surfaces during OER. Additionally, nanoscopic techniques are required to investigate the dissolution and redeposition behaviors of 3d transition‐metal cations as such dissolution or redeposition creates a few nanometer thick depth profiles or redeposited zones locally. A better understanding of how dissolution and redeposition processes reconstruct the surface is crucially important for pinpointing the mechanisms responsible for the activation or deactivation of electrocatalysts.

## Conflict of Interest

The authors declare no conflict of interest.
